# Defining the transcriptomic landscape of the developing enteric nervous system and its cellular environment

**DOI:** 10.1186/s12864-017-3653-2

**Published:** 2017-04-12

**Authors:** Sweta Roy-Carson, Kevin Natukunda, Hsien-chao Chou, Narinder Pal, Caitlin Farris, Stephan Q. Schneider, Julie A. Kuhlman

**Affiliations:** 1 Department of Genetics, Development and Cell Biology, Iowa State University, Ames, IA 50011 USA; 2 Present Address: National Cancer Institute, US National Institutes of Health, Bethesda, Maryland USA; 3 Present address: North Central Regional Plant Introduction Station, 1305 State Ave, Ames, IA 50014 USA; 4 Present address: Pioneer Hi-Bred International, Johnson, IA 50131 USA; 5 642 Science II, Iowa State University, Ames, IA 50011 USA

**Keywords:** Enteric nervous system, Neural crest, Transcriptome, RNA-sequencing, Zebrafish, Hirschsprungs, *phox2b*

## Abstract

**Background:**

Motility and the coordination of moving food through the gastrointestinal tract rely on a complex network of neurons known as the enteric nervous system (ENS). Despite its critical function, many of the molecular mechanisms that direct the development of the ENS and the elaboration of neural network connections remain unknown. The goal of this study was to transcriptionally identify molecular pathways and candidate genes that drive specification, differentiation and the neural circuitry of specific neural progenitors, the *phox2b* expressing ENS cell lineage, during normal enteric nervous system development. Because ENS development is tightly linked to its environment, the transcriptional landscape of the cellular environment of the intestine was also analyzed.

**Results:**

Thousands of zebrafish intestines were manually dissected from a transgenic line expressing green fluorescent protein under the *phox2b* regulatory elements [*Tg(phox2b:EGFP)* ^*w37*^]. Fluorescence-activated cell sorting was used to separate GFP-positive *phox2b* expressing ENS progenitor and derivatives from GFP-negative intestinal cells. RNA-seq was performed to obtain accurate, reproducible transcriptional profiles and the unbiased detection of low level transcripts. Analysis revealed genes and pathways that may function in ENS cell determination, genes that may be identifiers of different ENS subtypes, and genes that define the non-neural cellular microenvironment of the ENS. Differential expression analysis between the two cell populations revealed the expected neuronal nature of the phox2b expressing lineage including the enrichment for genes required for neurogenesis and synaptogenesis, and identified many novel genes not previously associated with ENS development. Pathway analysis pointed to a high level of G-protein coupled pathway activation, and identified novel roles for candidate pathways such as the Nogo/Reticulon axon guidance pathway in ENS development.

**Conclusion:**

We report the comprehensive gene expression profiles of a lineage-specific population of enteric progenitors, their derivatives, and their microenvironment during normal enteric nervous system development. Our results confirm previously implicated genes and pathways required for ENS development, and also identify scores of novel candidate genes and pathways. Thus, our dataset suggests various potential mechanisms that drive ENS development facilitating characterization and discovery of novel therapeutic strategies to improve gastrointestinal disorders.

**Electronic supplementary material:**

The online version of this article (doi:10.1186/s12864-017-3653-2) contains supplementary material, which is available to authorized users.

## Background

The enteric nervous system (ENS), the largest division of the peripheral nervous system (PNS), is composed of a network of neurons and glia that innervate the gastrointestinal tract [1]. The ENS functions within the gastrointestinal tract to control motility for mixing and moving food, absorption of nutrients, epithelial secretions, and blood circulation [2]. It arises from a population of multipotent neural crest cells (NCC), which migrate from the vagal, and in some species sacral, axial area to the intestine. There they continue to proliferate and differentiate into enteric neurons and glia within the gastrointestinal wall [3, 4]. The migration, specification and differentiation of the enteric neural crest are determined by intrinsic and extracellular cues. Thus, the enteric neural crest cells interact with other cells and respond to the local environments within the gut. Defects in the migration, proliferation or differentiation of the neural crest progenitors have been shown to lead to enteric neuropathies and are implicated in a variety of gastrointestinal dysmotility disorders [5–10].

Previous studies have identified many important genes and pathways required for normal ENS development [1, 6, 11–13]. Best understood are genes whose role in ENS development have been elucidated by loss-of-function studies or the study of congenital birth defects [14–23]. These genes encode a variety of transcription factors, cell-cell signaling molecules, neuropeptides, biosynthesis enzymes, cell adhesion molecules, soluble growth factors, extracellular matrix and cytoskeletal proteins. Categories of genes functionally linked to ENS development have been expertly collated and described in a number of recent reviews [10, 16, 24–26].

Among the most studied genes in ENS development, are the genes required for the Ret signaling pathway. Mutations in the RET tyrosine kinase receptor gene, it’s co-receptor GDNF Family Receptor Alpha 1 (GFRα1), and it’s ligand GDNF emerged as the most common causes of Hirschprung Disease (HSCR). In HSCR patients, insufficient proliferation and migration of enteric neural crest lead to partial loss of the ENS generally characterized by a complete lack of enteric neurons within the caudal region of the gastrointestinal tract [27–31]. The Ret signaling pathway is most commonly affected in HSCR, [1, 32] accounting for 20–50% of familial and 15–30% of sporadic cases [12, 16, 24]. The etiology of HSCR has recently been extensively covered in several reviews [16, 24, 25, 31, 33, 34]. In addition to Ret, more than a dozen other genes including DNA (Cytosine-5-)-Methyltransferase 3 Beta (DNMT3β), Endothelin Converting Enzyme 1 (ECE1), Endothelin 3 (EDN3), Endothelin Receptor Type B (EDNRB), Glial Cell Derived Neurotrophic Factor (GDNF), GFRα1, Kinesin Family Member 1B (KIF1B/KIAA1279), L1 Cell Adhesion molecule (L1CAM), Neurotrophin 3 (NTF3), Neuregulin 1 (NRG1), Neuregulin 3 (NRG3), Neurturin (NRTN), Neurotrophic Receptor Tyrosine Kinase 3 (NTRK3), PHOX2B, Prokineticin 2 (PROK2), Prokineticin Receptor 1 (PROKR1), Prokineticin Receptor 2 (PORKR2), Persephin (PSPN), Semaphorin 3A (SEMA3A), Semaphorin 3D (SEMA3D), SRY-Box 10 (SOX10), Transcription Factor 4 (TCF4), and Zinc Finger E-Box Binding Homeobox (ZEB2) are known to be associated with, or cause HSCR [10, 16, 31, 35–42]. Yet, the exact genetic cause of 50% of HSCR cases remain unknown [31].

As the enteric neural progenitors migrate into the gut, they proliferate and differentiate into at least 15 different neuronal subtypes that differ in their function, physiology, morphology, and neurotransmitter expression [43]. In mammals, these subtypes form ganglia that are organized into a network of two interconnecting plexi. The myenteric plexus is embedded between the circular and longitudinal smooth muscle layers, and the submucosal plexus is situated between the muscle and the mucosal layer. Each neuronal subtype expresses different combinations of neuropeptides giving them a unique neurochemical code [44, 45]. Along the length of the intestinal tract, the combinations of neuronal subtypes vary between the different regions of the gut [43]. Although the diversity of enteric neuronal subtypes and their neurochemical code has been well described, our understanding of the genes required for enteric neuronal specification, differentiation and for establishing neural connectivity within the ENS remains rather limited [30, 43, 46].

To fill this gap we pursued a broad strategy to identify candidate genes required to drive neural crest precursors to differentiate into enteric neurons including potential signaling molecules for the formation of neuronal subtypes and their integration into a functional neural network in zebrafish. Zebrafish embryos and larvae have emerged in recent years as a powerful complementary vertebrate model for ENS development. Although some important anatomical differences to the ENS in mammals exist e.g. the neurons and glia of the zebrafish myenteric plexus do not form ganglia [47] and the submucosal plexus is absent, the signaling pathways and genes associated with ENS development remain conserved [47–50]. Markers of differentiated enteric neurons can first be detected in the developing intestine approximately 48 hours post fertilization (hpf). Over the next 24 hours, neural crest cells continue to migrate and proliferate and by 74 hpf the entire length of the gut is colonized. The first contractions within the intestine are seen at 4 days post fertilization (dpf) and by 5 dpf the larval gut is functional and the larvae are capable of feeding [49, 51]. In this study, 7 days post fertilization (dpf) larvae from a transgenic zebrafish line were used to isolate enteric neuronal cells from the intestine. 7dpf represented the time in development at which a subset of neurons has fully differentiated; yet the ENS is not fully developed and likely to retain some precursors.

To track and distinguish precursor cells and differentiating enteric neurons we made use of a zebrafish transgenic line in which EGFP is expressed under the regulatory/promoter region of the transcription factor *phox2b, Tg(phox2b:EGFP)* ^*w37*^ [52]. This strategy was chosen because *phox2b* is required for the specification and development of the neural crest-derived autonomic nervous system including the enteric neurons and glia in various model systems [53–55]. Loss of phox2b during development leads to loss of enteric ganglia in mice [54] while in heterozygous animals a normal GI tract is observed [5, 16, 56, 57]. In humans, mutations in PHOX2B are linked to a loss of enteric neurons in the gastrointestinal tract leading to HSCR, and many cases have been associated with congenital central hypoventilation syndrome [5, 56, 58, 59]. Determination of the genetic processes underlying the syndrome revealed that mutations of the PHOX2B gene are principally responsible for the broad range of symptoms encountered by disrupting early development of autonomic neurons [56, 60–63]. Importantly, phox2b has been shown to regulate Ret expression [64, 65] and the genetic interaction between Ret and phox2b has been demonstrated to be critical for normal ENS development [16, 54, 56, 57]. In mice phox2b expression is turned on as the neural crest cells (NCCs) leave the neural tube, remains expressed as neural crest migrate into the intestine and differentiate into neurons and glia and continues to be expressed in ENS derivatives into adulthood [55].

Our goal was to generate a comprehensive transcriptional profile of enteric neurons along the entire intestinal tract during normal development. To improve the chances of obtaining discrete transcript counts and unbiased detection of novel or low-abundance transcripts across a broader dynamic range [66], endogenous enteric neurons and enteric neural crest progenitors were transcriptionally profiled using RNA-seq. Complimentary to profiling the enteric neurons, we also utilized RNA-seq to transcriptionally profile their surrounding non- neural microenvironment [67], the intestine including the mucosa, musculature and associated vasculature. Thus, this strategy enabled us to perform a comparative gene expression study between the neuronal cell and the non- neuronal cell population of the intestine. Our analyses identified scores of candidate genes that may function as cell fate determinants during ENS development, may constitute useful markers to distinguish different ENS subtypes, and define the surrounding cellular microenvironment of the ENS on a molecular level. A subset of the genes has been validated by expression studies, and selected for ongoing functional studies. The result of these studies will not only expand our knowledge of genes required for normal ENS development and a better understanding of the cellular and molecular basis of the complex process of development of the ENS but may help inform stem cell therapies aimed at directing lineage specific enteric neuron development.

## Methods

### Animal husbandry and line

The embryos and larvae were obtained from a transgenic zebrafish line as described previously and provided by Dr. A Nechiporuk (Oregon Health Sciences), in which green fluorescent protein (GFP) is expressed under the regulatory elements of the transcription factor *phox2b Tg(phox2b:EGFP)* ^*w37*^ [52]. The embryos and larvae were raised at 28.5 °C in a light/dark cycle according to standard zebrafish husbandry protocols and staged to seven days post fertilization (d.p.f) [68]. Larvae were prescreened for GFP expression and anesthetized prior to dissection. All animal experiments were performed in accordance with the U.S. laws, guidelines and policies of laboratory animal care and use and approved by the Institutional Animal Care and Use Committee and Institutional Biosafety Committee at Iowa State University.

### Tissue collection and RNA isolation

To acquire the enteric neuronal population, larvae from the transgenic line *Tg(phox2b:EGFP)* ^*w37*^ were dissected at 7 days post fertilization (dpf) to isolate the entire gut. Guts were dissociated into cells using papain digestion. For dissociation, 20 μl of 41.2mgP/ml papain was added to the dissected guts along with 360 μl of a solution of HBSS, 0.01% of HEPES and 20 μl of an activation agent according to the manufacturer’s instructions (Worthington cat #LS003126). The samples were incubated at 37 °C for a total of 30 minute with trituration of the sample every 10 minutes. A final incubation was done for 13 minute with 20 μl of DNase I. The samples were resuspended in 500 μl of 0.1% BSA in PBS. Fluorescent activated cell sorting (FACS) was done on each sample using a BD FACSAria III available at the Iowa State Flow Cytometry facility. GFP-positive cells were detected through a 525/50 nm bandpass filter, and simultaneously checked for viability, and sorted directly into lysis buffer (Additional file 1: Figure S1). Both, the GFP positive neuronal cells and the GFP negative non-neuronal cells were collected directly into commercially available lysis buffer and stored at -80 °C until separated cell populations from multiple FACS sorting experiments could be pooled and processed. RNA isolation was done using pooled samples of 100,000–255,000 cells and the Ambion™ ‘s RNAqueous®- Micro total RNA isolation kit. The protocol was followed as per kit instructions with the following modifications. A total volume of 20 μl of elution buffer was used and the column was incubated at room temperature for 10 minutes before the final spin to collect the eluted RNA. A total of 4795 larval intestines were dissected to collect 373,749 GFP positive cells to obtain sufficient RNA for two biological replicates of the GFP-positive cell population. 400,000 GFP negative cells were collected from the same 4795 larval intestine samples for the two biological replicates of the GFP-negative cell population. The RNA quality check was checked using an Agilent 2100 Bioanalyzer. RNA integrity number (RIN)s of 8.4-8.5 were measured for all RNA samples. Libraries were prepared using Illumina’s TruSeqv2 kit (cat#15026495).

### Sequencing and raw read processing

The sequencing was done using an Illumina HiSeq 2500 sequencing system at the DNA facility of the Iowa State University office of Biotechnology. Deep sequencing of the mRNA was performed with 100 bp paired-end reads. Each biological replicate was considered as a separate sample. Preprocessing of the raw reads was done using Trimmomatic, [69] to remove adapters and the low quality reads. The preprocessing was done using the parameters LEADING:15 TRAILING:15 SLIDINGWINDOW:4:20 MINLEN:36. The removal of the bases was based on the Phred score where the threshold was 20. A window size of 4 was used which removed the bases with an average Phred score of less than 20. The quality of the trimmed reads was analyzed and visualized using FASTX-Toolkit [70].

### Alignment, transcript abundance and differential gene expression analysis

The trimmed reads were mapped to the Zv9 *Danio rerio* reference genome using Tophat2 [71] which uses Bowtie2 [72] as the mapping tools. The version Zv9.76 of GTF (gene transfer format) was used for annotation of the mapped reads. The expression levels of the mapped reads were obtained using featureCounts [73] to specifically count the transcripts mapping to exons. For the analyses shown in Figs. 2 and 3a and b, we used Cufflink to get the Fragments Per Kilobase of transcript per Million mapped reads (FPKM) [74]. An R package DESeq [75] was used to perform the differential gene expression analysis between the GFP-positive enteric neuron gut population and the GFP-negative non-neuronal gut population. The software considered the biological replicates from both the cell populations during the analysis to provide an expression level for the genes. The differentially expressed genes were filtered using the False Discovery Rate (FDR) adjusted *p-value* ≤ 0.01. The significantly upregulated and downregulated genes were chosen based on a log2foldchange value of 1.5. The heatmap was generated using the R function “pheatmap”. For the correlation analysis, the Spearman correlation coefficient for pairwise samples was calculated using R function “cor”. The scatter plots were visualized using R function “plot”. The expression value was log-transformed using log2 (FPKM + 1).

### Gene Ontology enrichment and Pathway analysis

A Gene Ontology (GO) enrichment analysis was performed using the cytoscape plugin BiNGO [76] with the adjusted *p*-value < 0.05 where the FDR correction was done by Benjamin and Hochberg’s method. Pathway analysis was performed to identify pathways most enriched in the enriched and diminished genes in the neuronal population using QIAGEN’s Ingenuity Pathway Analysis (IPA) (IPA®, QIAGEN Redwood City, www.qiagen.com/ingenuity). To perform IPA pathway analysis human and mouse orthologues of the identified Zebrafish Ensembl genes were converted and IPA pathway analysis was performed on 44% of the genes from the complete data set. The datasets analyzed were the list of significantly enhanced genes; and the list of significantly diminished genes. The percentage of molecules was calculated for the analysis using a total of 1133 genes from the enhanced list and a total of 696 genes from the depleted list.

### qPCR

Eight candidate genes were chosen for qPCR. qPCR was performed on the same cDNA libraries used for Illlumina sequencing to validate the RNAseq results. Primers were designed using NCBI primer designing tool (http://www.ncbi.nlm.nih.gov/tools/primer-blast/) and synthesized at the Iowa State DNA facility (http://www.dna.iastate.edu/). Primers to *chn1, elavl3, chata, phox2bb, syn2a, amy2a, fli1a*, and *hprt1* were designed to span exon-exon junction and give a single product of 150–200 bp. Individual qPCR reactions were carried out with the standard StepOnePlus (Applied Biosystems™) cycling protocol using PerfeCTa SYBR® Green FastMix, ROX (Quantabio) according to manufactures instructions. For each qPCR run, the Cycle threshold (Ct) were exported into Microsoft excel for statistical analysis. We used 2^-ΔΔCt^ method of relative quantification to estimate copy number of selected genes [77]. The expression levels were normalized to zebrafish (*Danio rerio*) hypoxanthine phosphoribosyltransferase 1 (*hprt1*) as a reference gene [78].

## Results

### Transcriptional profiling of ENS cells and the zebrafish intestine

**Fig. 1:**
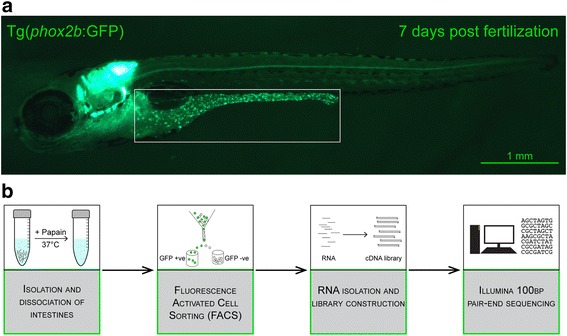
Image and flow chart illustrating source of GFP labeled cells, sample preparation and bioinformatics analysis. **a** Image of a 7 days post fertilization (dpf) zebrafish larvae of the transgenic line, *Tg(phox2b:EGFP)*. The boxed area shows the intestine with GFP-positive enteric neurons (*green dots*). **b** Schematic diagram illustrating the experimental approach. Intestines were dissected out to avoid contamination with GFP-positive cells expressing *phox2b* in the hindbrain or the spinal cord. The intestines were dissociated using papain digestion followed by fluorescent activated cell sorting (FACS). RNA isolated from the sorted cell populations was used to construct cDNA libraries. Raw reads from Illumina sequencing were processed and analyzed using various bioinformatics analyses programs

To identify genes required for the specification and differentiation of enteric neurons from neural crest progenitors we isolated and carried out RNA-seq transcriptional profiling on a cell lineage expressing the transcription factor *phox2b. phox2b* has been shown to be expressed in early enteric neural crest progenitors as they migrate into the gut [54]. As development proceeds *phox2b* expression remains high in a subset of the differentiated neurons into adulthood and decreases to lower levels in the developing glia [55]. *phox2b* expression is retained in a subset of adult ENS derivatives. Therefore, a transgenic reporter line driving expression of GFP under the *phox2b* promoter, *Tg (phox2b:EGFP)* ^*w37*^ [52] seemed a good candidate line to identify enteric neuron precursors and enteric neurons as GFP-positive cells within the intestine, and to facilitate separation of the neuronal from non-neuronal cell populations of the intestine for subsequent transcriptional profiling. Indeed, personal observations confirmed that GFP expression appears in the enteric neural crest as they migrate into the intestine and continues throughout development and into adulthood in the zebrafish *Tg(phox2b:EGFP)* ^*w37*^ line (Fig. 1a). Thus, GFP-positive cells of the intestine mark both enteric neurons and enteric neuron precursors in 7 days post fertilization (dpf) larvae and may facilitate separation of the ENS cells from other intestine tissues by fluorescence activated cell sorting (FACS). However, GFP expression is not restricted to ENS in 7 dpf larvae. Endogenous *phox2b*, and therefore GFP, in the zebrafish *Tg(phox2b:EGFP)* ^*w37*^ line is also expressed in cells of the hindbrain and cranial ganglia as seen in Fig. 1a. To ensure that only GFP-positive cells (GFP^+^) that comprise the ENS were profiled, the intestinal tracts of 7dpf zebrafish larvae were manually dissected away from the remaining larval body prior to dissociation and FACS sorting (see Methods for details).

At this stage in development, the enteric neuron precursors have migrated to the end of the intestine, and a sufficient number of progenitors have differentiated into different ENS subtypes to form a functioning ENS. Enteric neurons present at this time reflect at least some of the diversity of enteric neuron subtypes [45]. After enzymatically treating the dissected intestines with papain, the dissociated cells were FAC sorted and viable GFP positive cells were collected (Additional file 1: Figure S1). Cells from whole intestines of wild type zebrafish were used as a negative control to establish a threshold for GFP expression. Viable GFP-negative cells representing the remaining cell types in the intestine were collected simultaneously and processed in the same manner as the GFP-positive cells. To minimize potential changes in gene expression patterns, to obtain sufficient amounts of RNA to sequence, and to limit amplification steps, dissection times were limited and multiple independent cell sorts were pooled. A total of 4795 larval intestines were dissected to collect 373,749 GFP positive cells to obtain sufficient RNA for two biological replicates of the GFP-positive cell populations. 400,000 GFP negative cells were collected from the same larval intestine samples for the two biological replicates of the GFP-negative cell populations. Libraries were generated from the pooled samples using unamplified RNA. Biological duplicates of both the GFP-positive (neuronal) and GFP-negative (non-neuronal) cells were sequenced using Illumina HiSeq 2500. Following sequencing, a developed pipeline was implemented to process the raw RNA-seq reads (see Methods). Figure 1b outlines the basic workflow from tissue collection to data processing.

### Differential gene expression analysis between neuronal and non-neuronal cells of the intestine

To identify genes that may be required for the differentiation and assembly of enteric neuron circuitry we looked for differences in expression profiles between the sorted neuronal (GFP+) and non-neuronal (GFP-) populations of intestinal cells. For differential gene expression analysis the raw reads were trimmed using Trimmomatic to remove the adapters and the low quality reads, and mapped to the annotated *D.rerio* reference genome (Zv9) (see Methods for further detail).

**Fig. 2:**
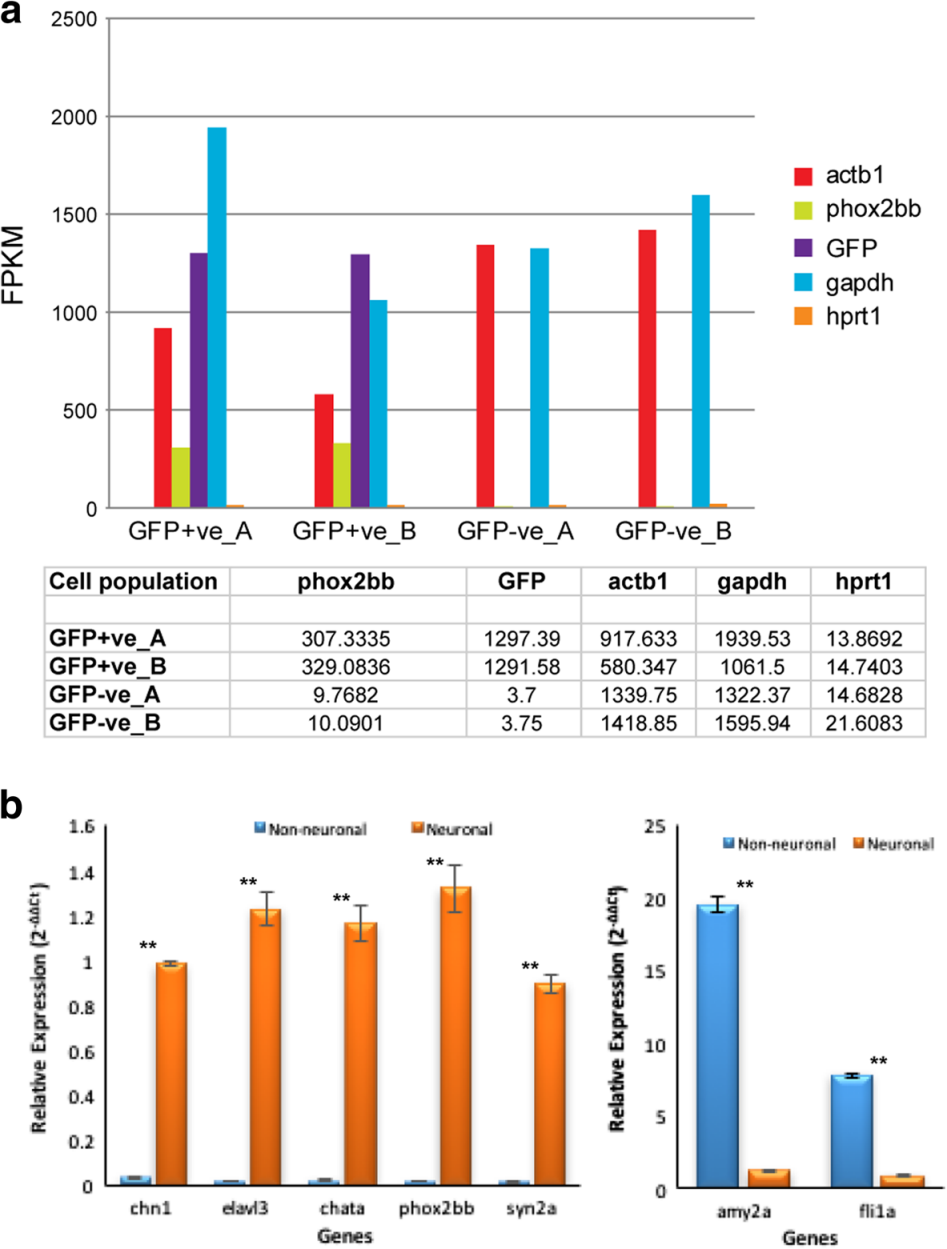
Experimental validation of reliable separation of the neuronal ENS from the non-neuronal intestinal cell population. **a** The bar graph (above) and corresponding data in table (below) show the expression levels in FPKM of five selected genes in the two neuronal replicate [GFP-positive_(GFP + ve) **a** and _**b**] and the two non-neuronal replicate [GFP-negative_(GFP-ve) **a** and _**b**] cell populations. GFP transcript levels show the level of GFP transcript driven from the *phox2b* transgene. The expression values of p*hox2bb* measure the endogenous transcript levels of the *phox2b* gene. Expression levels for the three house-keeping genes *actb1, gapdh*, and *hprt1* are also shown. (**b**) qPCR validation of expression levels of 7 genes comparing the GFP-positive neuronal populations (*Orange bars*) versus the GFP-negative non-neuronal (*blue bars*). *chn1, elavl3, chata, phox2bb,* and *syn2a* were more highly expressed in the neuronal population, whereas *amy2a* and *fli1a* were more highly expressed in the non-neuronal population. Changes in gene expression were calculated using 2^− ΔΔCT^ method with *hprt1* as the house- keeping gene. The mean relative expression of the neuronal population was significantly different from that of the non-neuronal population (*p*-value < 0.01)

To validate that our experimental approach separating the ENS/neuronal cells from non-neuronal cells by dissociation and FAC sorting, and that our differential gene expression analysis yielded meaningful results, we tested for several predicted outcomes. First, GFP mRNA should be entirely restricted to the two neuronal GFP-positive cell samples and absent in the two non-neuronal GFP-negative samples. Indeed, high levels of GFP (~1300 FPKM) were found in the two neuronal, and miniscule amounts (<4FPKM) in the two non-neuronal samples (Fig. 2a). Therefore, dissociation and sorting successfully separated the two intestinal cell populations leading to a >300 fold enrichment of GFP in the neuronal versus non-neuronal samples. Second, we would expect that endogenous *phox2b* mRNA should also be enriched in the GFP-positive cells. Indeed, endogenous *phox2b* was highly enriched in the GFP-positive cells (>300 FPKM), a ~30 fold enrichment compared to the GFP-negative samples while high and low expressing house-keeping genes like *actb, gapdh*, and *hypoxanthine phosphoribosyltransferase 1 (hprt1)* showed similar high and low expression in both sorted cell populations. This demonstrates that GFP expression in the intestine in the zebrafish *Tg(phox2b:EGFP)* ^*w37*^ line is indeed labeling the *phox2b*-expressing ENS cell population.

To verify that our estimated abundance levels for transcripts by RNA-seq are consistent with the actual expression levels in our samples we determined the relative abundance levels of a few candidate genes using semi-quantitative PCR (qPCR). Based on our experimental strategy we would predict that candidate genes that are known to control ENS development and differentiation are entirely expressed or strongly enriched in the GFP-positive cells. Conversely, we would expect that that known genes involved in patterning and differentiation of the non-neuronal intestine should be strongly enriched in the GFP-negative samples.

Indeed, relative expression levels of the candidate genes using semi-quantitative PCR (qPCR) correlated with the RNAseq results, and showed the predicted differences in both cell populations (Fig. 2b). *ELAV like neuron-specific RNA binding protein 3 (elavl3)*, a RNA binding protein found in post mitotic neurons and specific only to neurons, *choline O-acetyltransferase a (chata),* whose gene product is required for biosynthesis of the neurotransmitter acetylcholine, *phox2bb*, a DNA-binding protein which is associated with the development of noradrenergic neurons, *Chimerin 1 (chn1)*, a gene that is primarily expressed in the neurons and is involved in neuronal signal-transduction and *synapsin 2a (syn2a)*, a synaptic gene that has been involved in neurotransmitter release and synaptogenesis, were all found to be highly expressed in the neuronal population consistent with their roles in neurogenesis and synaptogenesis. Whereas *amylase alpha 2a (amy2a)*, one of the genes that is highly involved in proper digestion by helping in breakdown of polysaccharides and oligosaccharides and Fli-1 proto-oncogene*, ETS transcription factor a (fli1a),* showed higher levels in the non-neuronal cell population. *hrpt1* was chosen as a more reliable house keeping gene [78]. Our results showed that there was a high correlation of relative expression levels between the RNA-seq results and the qPCR for all eight genes. Furthermore, the expression for both groups of marker genes are almost entirely separated demonstrating that the ENS, and the non-neuronal cell populations have been efficiently separated by dissociation and sorting with a minimum of cross contamination.

All of the above indicates that a genome scale differential expression analysis between GFP-positive and GFP-negative cell populations will yield meaningful transcriptional profiles of neuronal/ENS cells and non-neuronal cells of the zebrafish intestine, respectively. To do so we compared the genes expressed in the neuronal and non-neuronal cell populations of the intestine. 4418 differentially expressed genes (DEGs) were identified (where *n* = 2, False Discovery Rate (FDR) by Benjamin and Hochberg’s test ≤ 0.01 and log2foldchange ≥ 1.5) (Additional file 2: Table S1). Of these DEG’s, 2561 genes were found to be significantly enriched in the neuronal cell population (Additional file 2: Table S1). For ease in this manuscript, we refer to genes expressed at significantly higher levels in the neuronal population in comparison to the non-neuronal population as ‘enriched’ and to genes most significantly lower in the neuronal population as ‘depleted’.

**Fig. 3:**
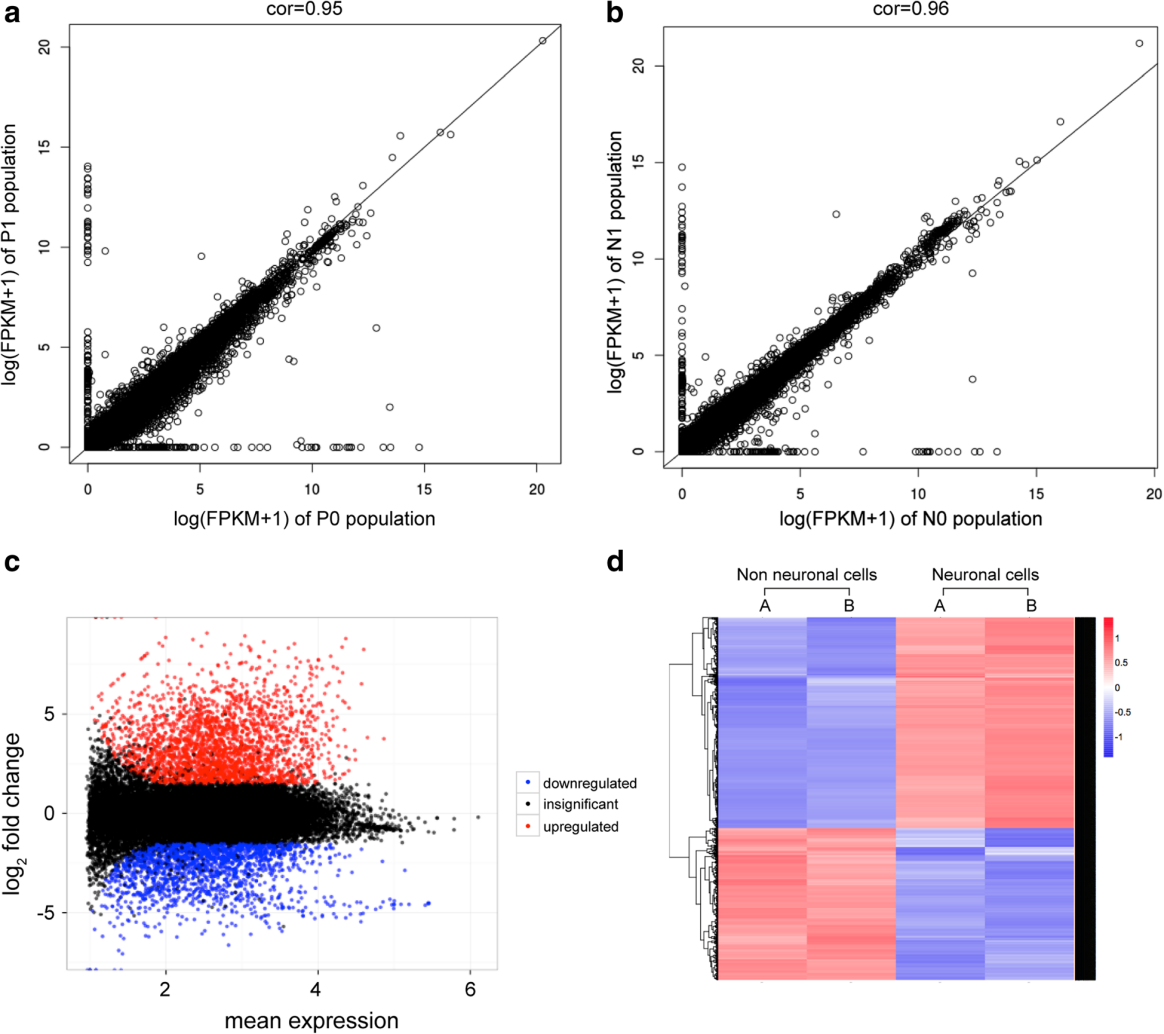
Differential gene expression analysis of 7dpf *Danio rerio* enteric neurons. **a** and **b**. Plot showing the correlation between the two biological replicates of the GFP-positive neuronal population (**a**) and of the GFP-negative non-neuronal population (**b**). The x-axis represents the 1^st^ biological replicate while the y-axis represents the 2^nd^ biological replicate. The correlation coefficient was calculated using FPKM. The correlation coefficient for the neuronal and non-neuronal cell population is 0.95 and 0.96, respectively. **c** Dispersion plot showing the log2foldchange of individual reads as compared to the mean normalized counts using DESeq with the adjusted *p-value* ≤ 0.01. The *red dots* represent the upregulated/enriched and the *blue dots* represent downregulated/depleted genes in the neuronal cells of the zebrafish intestine. **d**. Heat map of the normalized counts for the 4418 DEGs between neuronal and non-neuronal cell populations. The thresholds selected are FDR ≤ 0.01 and log2foldchange ≥ 1.5. The first two columns represent biological replicates (**a** and **b**) of the non-neuronal cell population and the second two columns represent biological replicates (**a** and **b**) of the neuronal population. Each row denotes a single gene and their expression pattern across the two different samples. The *blue regions* in the neuronal population represent the 57.99% of the upregulated genes and the *red region* represents the downregulated genes. The dendogram on the x-axis shows the grouping of same samples (biological replicates) while the y-axis shows clustering of (genes with) similar expression patterns

Both the neuronal and non-neuronal cell populations showed a high level of similarity in the FPKMs between duplicates prompting us to expand our analysis to compare all the genes expressed above FPKM of 1 in our data sets. To analyze how reproducible the estimated gene expression levels between the biological duplicates were, we calculated the Spearman Correlation coefficient for all expressed genes (Fig. 3a and b). A comparison of the biological replicates of the neuronal and non-neuronal cells yielded average coefficients of 0.95 and 0.96 respectively demonstrating a high correlation of gene expression levels between the duplicates (Fig 3a and b). The high level of concordance between biological replicates confirms the quality and consistency of our samples and dataset.

To illustrate the differentially expressed genes and their expression pattern visually, we generated a dispersion plot (Fig. 3c) heat map (Fig. 3d) showing the clustering of differentially expressed gene expression profiles for the neuronal and non-neuronal samples (Fig. 3d shows the heat map of the normalized counts, see Additional file 2: Table S1 for the complete list). The heat map shows the relative expression of the genes based on their mapped read counts. Enriched genes include genes such as *phox2b, elavl3, ret* and *gng3*, previously associated with ENS development [1, 79]. Interestingly, HSCR related genes such as *gfra1 (a and b), ret, phox2b*, and *zeb2* that cause aganglionosis when knocked down in zebrafish or mutated in mice are also found enriched [11]. Other elevated genes in the neuronal cell population are known to impact the ENS functionally without aganglionosis including *ascl1, cadherin EGF LAG seven-pass G-type receptor 3 (celsr3), frizzled class receptor 3 (fzd3), gfra2, hand2, hoxb5 (a and b), kinesin family members (kif26 (a and b)), neural adhesion molecules (nadl1.1, nadl1.2), ntrk3 (a and b), solute carrier family 6 member 2 (slc6a2), T-cell leukemia homeobox 2 (tlx2)* [6, 10, 11]*.* Among the genes significantly depleted in the neuronal cell population, we find genes that lead to intestinal aganglionosis *endothelin converting enzyme 1 (ece1), Indian hedgehog homolog a (ihha), integrin, beta 1b.1 (itgb1b.1), itgb.2*, and genes that cause alterations in ENS function like *distal-less homeobox 2a (dlx2a), erb-b2 receptor tyrosine kinase 3a (erbb3a), noggin (nog1, 2 and 3), sonic hedgehog b (shhb), and zic family member 2 (zic2 (a and b)* [1, 80]. Therefore, through the DE analysis we were able to identify previously characterized as well as many novel genes that are specifically expressed in the neuronal versus non-neuronal cell population verifying the quality of our samples and datasets. These findings suggest that the identified enriched and depleted genes constitute a comprehensive and valuable resource for discovery and subsequent targeted functional analysis of enteric neuronal and non-neuronal cell types within the intestine, respectively.

### GO and Molecular enrichment analysis within the two intestinal cell populations

To identify specific functional, molecular as well as structural gene categories within the enriched or depleted gene sets, a Gene Ontology (GO) enrichment analysis was performed. Among genes associated with biological processes, 143 GO terms were enriched within the GFP-positive, neuronal cell population, while 280 GO terms were enriched among genes within the GFP-negative, non-neuronal cell population. We generated comprehensive GO term hierarchies where each node represents the number of genes associated with a particular GO term, and the intensity of the orange to yellow to white color represents the significance from high to low, respectively. Biological process hierarchies were generated for both neuronal [Additional file 3: Figure S2 an interactive network file can be found in the BioGRID repository). Additional file 4: Figure S3 and Additional file 5: Figure S4 show the bargraphs of the 50 most significant GO terms represented in the neuronal population; based on *p*-value and percentage of genes respectively] and non-neuronal populations (Additional file 6: Figure S5 and. Additional file 7: Figure S6 show the bargraph of 50 most significant GO terms in the non-neuronal population). Similar analyses were performed for molecular function and cellular component GO categories in both the neuronal and non-neuronal populations (Additional files 8–11: Figures S7-S10 (molecular function category in neuronal population), Additional files 12–14: Figures S11-S13 (cellular component in neuronal population), Additional files 15–17: Figures S14-S16 (molecular function category in non-neuronal population), and Additional files 18–20: Figure S17-S19 (cellular components in non-neuronal population).

**Fig. 4:**
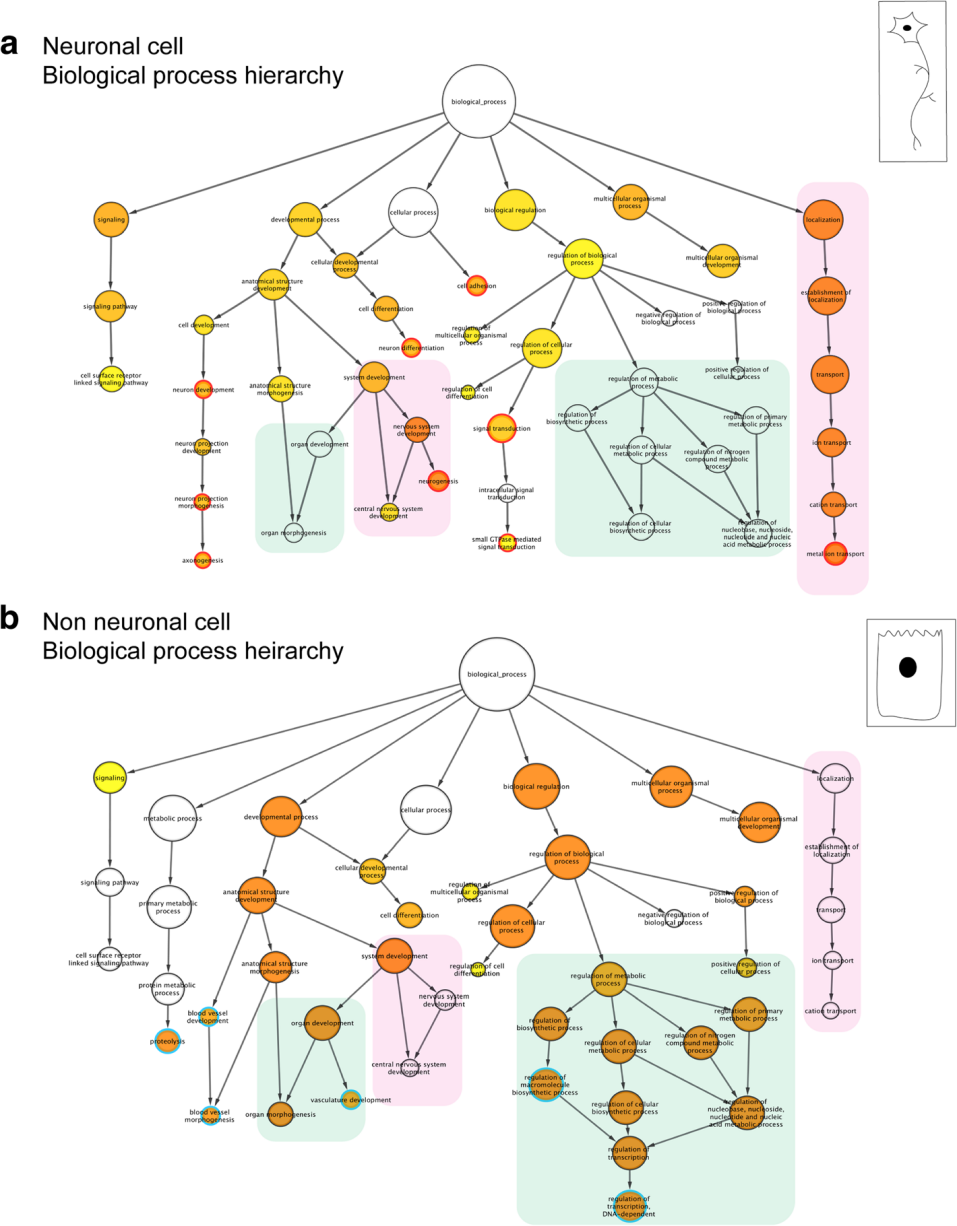
Gene Ontology (GO) enrichment analysis of the differentially expressed genes in the neuronal cells. GO enrichment analysis was performed using the enriched and the depleted genes from the neuronal cells. Figures illustrate a subset of GO terms common to both the neuronal and non-neuronal population biological properties hierarchies (see Additional files 9 and 18 for complete hierarchies, the interactive hierarchies can be obtained from the BioGRID repository) and a few specific to one or the other hierarchy (**a**) and (**b**). The hierarchies of the GO terms are based on the number of genes and the significance for the category biological process (compare to Table 1). The circles/nodes represent the different GO terms, where the size of the circle represent the number of genes associated with the term, i.e., the larger the circles, the more the number of genes associated with the GO term. The color scheme represents the significance: *white* represents the least, and *orange* represents the most significant terms. The *white*, *yellow* and *orange circles* represent the terms common to both the neuronal and non-neuronal networks, while the *red* and *blue* circumferences of the circles represent terms specific to either the neuronal or non-neuronal data sets respectively. **a** Core hierarchy of GO terms defining biological processes in enriched genes comprising the ‘neuronal’ gene set. 530 genes are included in this analysis. **b**. Core hierarchy of GO terms defining biological processes in the depleted genes comprising the ‘non-neuronal’ gene set. 482 genes are included in this analysis. The *green* and *pink boxes* highlight examples of sub hierarchies within the larger hierarchies of biological process GO terms common to both populations but differentially enriched between the two. Sub-hierarchies of biological process GO terms are significantly enriched in the neuronal population, ‘ion transport’ and ‘nervous system development’ but not in the non-neuronal population (shaded *pink boxes*). Examples of GO term sub-hierarchies enriched in the non-neuronal population include regulation of ‘metabolic and biosynthetic processes’ and ‘organ development’ (shaded *green boxes*). The corresponding *p*-values and percentage of genes associated with the GO terms in the *green* and *pink boxes* as well as the specific terms in either population are shown in Table 1

Some of the most significant GO term nodes were selected to generate a core hierarchy for easier visualization (Fig. 4). 40 terms found in both sets of analyses were included to illustrate the differences in gene categories between the neuronal and non-neuronal population. Interestingly the results show several of the GO terms and their associated genes have reciprocal expression patterns between the neuronal and non-neuronal populations. For example, the GO term ‘nervous system development’ is highly significant (see bright orange node) among genes enriched in the neuronal cell population with a *p*-value of 2.62e-10, while showing little or no significance in the depleted list (see white node) (pink boxes in Fig. 4). Conversely, terms like ‘organ development’ (7.38e-18) and ‘regulation of primary metabolic processes’ (4.46e-8) had low or no significance in the neuronal population but were highly significantly enriched in the non-neuronal set (green boxes in Fig. 4). The list for the selected GO terms present in the highlighted pink and green boxes as well as the terms specific to either population along with their *p*-values and percentage of associated genes corresponding to Fig. 4 can be found in Table 1. Additional file 21: Table S2 gives the *p*-value and percentage of genes for the additional top 50 GO term categories in the analyses.

**Table 1:** GO terms for selected nodes in the enhanced and depleted list

	Enhanced Genes		Depleted Genes	
Biological Processes	*p*-value	% of genes	*p*-value	% of genes
Axonogenesis	2.10E-06	2.8	--	--
Blood vessel development	--	--	4.35E-07	4.1
Blood vessel morphogenesis	--	--	9.14E-08	3.9
Cation transport	2.79E-13	8.4	<5.00E-2	--
Cell adhesion	4.01E-07	5	--	--
Central Nervous System development	1.42E-03	3.5	<5.00E-2	--
Establishment of Localization	1.45E-19	23.5	<5.00E-2	--
Ion transport	2.75E-20	12.2	<5.00E-2	--
Localization	2.13E-20	25.6	<5.00E-2	--
Metal ion transport	1.39E-12	7.1	--	--
Nervous system development	2.62E-10	8.6	<5.00E-2	--
Neurogenesis	2.74E-08	5.4	--	--
Neuron development	1.17E-06	3.5	--	--
Neuron differentiation	1.37E-06	4.3	--	--
Neuron projection morphogenesis	2.46E-06	2.8	--	--
Organ development	<5.00E-2	--	7.38E-18	18.8
Organ morphogenesis	<5.00E-2	--	9.16E-09	7.8
Positive regulation of Cellular process	<5.00E-2	--	4.13E-06	4.1
Proteolysis	--	--	2.53E-08	8
Regulation of Biosynthetic process	<5.00E-2	--	3.07E-09	17.4
Regulation of Cellular Biosynthetic process	<5.00E-2	--	2.70E-09	17.4
Regulation of Cellular Metabolic process	<5.00E-2	--	1.11E-08	18
Regulation of macromolecule Biosynthetic process	--	--	1.92E-09	17.4
Regulation of Metabolic process	<5.00E-2	--	2.61E-07	18.4
Regulation of Nitrogen compound Metabolic process	<5.00E-2	--	5.29E-09	17.4
Regulation of nucleobase, nucleoside, nucleotide & nucleic acid metabolic process	<5.00E-2	--	4.87E-09	17.4
Regulation of Primary Metabolic process	<5.00E-2	--	4.46E-08	17.8
Regulation of Transcription, DNA-dependent	--	--	2.70E-13	15.3
Signal transduction	1.19E-05	12	--	--
Small GTPase mediated signal transduction	3.56E-03	3.3	--	--
System development	6.88E-06	13.7	6.42E-15	19
Transport	6.16E-19	23.2	<5.00E-2	--
Vasculature development	--	--	2.25E-07	4.3

The ‘--‘ indicates N/A in the table

The terms specific to a single category have ' --' in both the, *p*-value and percentage of genes columns

In addition to shared GO terms found in both the enriched and depleted gene sets, several significant GO terms were identified belonging specifically to one set or the other. In the neuronal population the GO terms ‘neurogenesis’, ‘neuron development’, and ‘axonogenesis’ were highly significant as expected, but these GO terms were not present in the non-neuronal population (shown by nodes with red border in Fig. 4). Similarly, GO terms such as ‘proteolysis’, ‘vasculature development’ and ‘blood vessel morphogenesis’ were significantly enriched in the non-neuronal but absent in the neuronal cells (shown by nodes with blue border in Fig. 4). In addition, the most significantly enriched GO terms of the neuronal population emphasized the differentiation states and the establishment of neural circuitry between enteric neurons. Terms significantly enriched include terms relating to ‘synaptic function’ and ‘neurotransmission’, and are consistent with the onset of a functioning ENS at this stage in development.

**Fig. 5:**
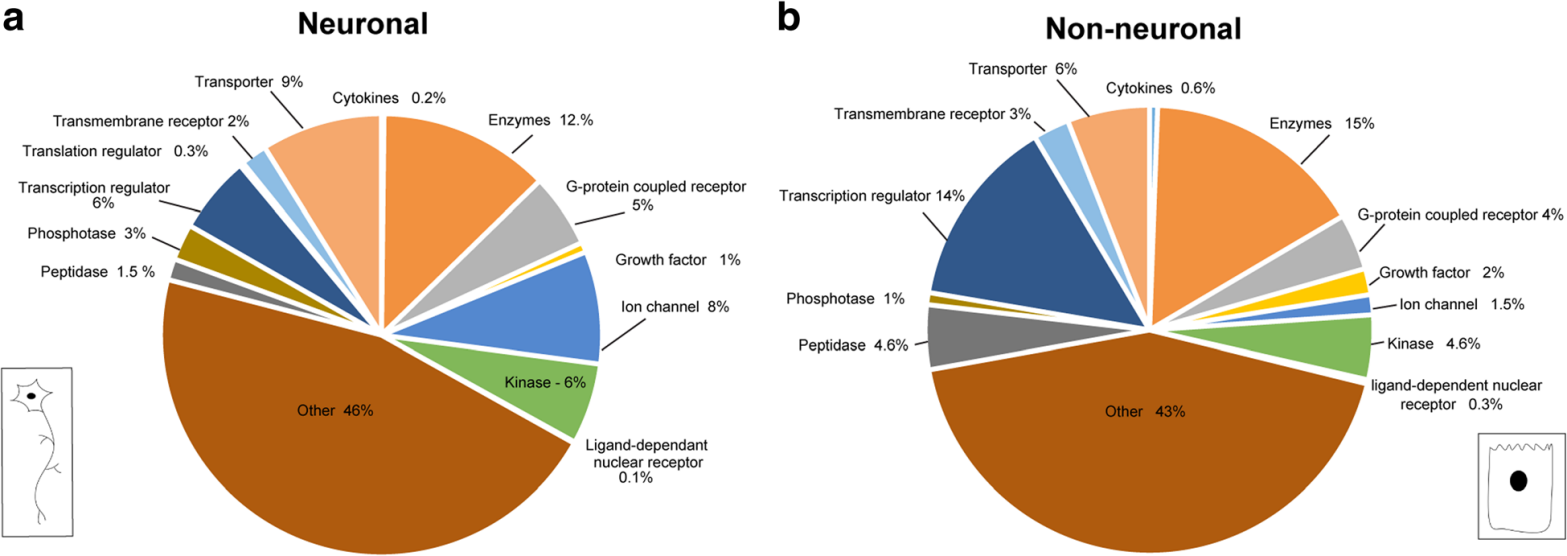
Gene function category enrichment of the significantly differentially expressed genes in the neuronal and non-neuronal gene populations: Pie-chart showing the most functionally enriched terms in the significantly differentially expressed genes IPA analysis. **a** gene function categories enriched in the neuronal population. **b** Gene category functions enriched in the non-neuronal population. Different colors represent different molecular functions and the size of the pie slice, the percentage of genes representing this category. Each category represents differentially expressed genes grouped by functional category. For example, ion-channels represent 8% of the DEG in the neuronal population (**a**) but only 1.5% of the genes in the non-neuronal population (**b**). The surprisingly high percentage of the genes are listed as ‘other’ because they are not represented within the functional categories defined by the IPA software parameters. Number of genes associated with each category is available in Additional file 22: Table S3

To gain further insights into the diverse functional molecular categories between the two cell populations, a differentially expressed gene (DEG) analysis was performed using QIAGEN’s Ingenuity Pathway Analysis (IPA) software. The identified functional categories and their percentage based on the total number of genes in each population are shown in the pie-charts in Fig. 5. This analysis has important limitations. The first pie-chart (Fig. 5) shows the categories of enriched genes according to their molecular function and their corresponding percentages within the neuronal population. Here, the total number of identified genes was 2561, of which 1205 were mapped to the IPA knowledge base, and 1133 were subsequently used for the analysis shown. The second pie-chart (Fig. 5b) shows a similar analysis in the depleted genes, with an initial input of 1844 genes, of which 751 were mapped and 696 were found to be analysis ready. Therefore, of the 2383 gene orthologues included in the analysis 1459 genes were assigned to functional designated categories. The remaining mapped genes, though equally interesting, could not be formally assigned to a functional category due to limitations in IPA category designations and were classified as ‘others’ (Additional file 22: Table S3). When the molecular categories within the enhanced and the depleted genes were examined separately, the neuronal population showed 8% ‘ion-channels’ as compared to 1% in the non-neuronal population. In contrast, there were 5% ‘peptidases’ in the non-neuronal population as compared to 2% in the neuronal population. Additionally, other categories such as ‘growth factors’, ‘transcriptional regulators’ and ‘cytokines’ were enriched in the non-neuronal population (2%, 14% and 1%, respectively) as compared to the neuronal population (1%, 6% and 0.2%, respectively) (Fig. 5). Thus, this analysis categorized genes in both cell populations based on their association with biological or molecular functions indicating significant shifts in several categories and identifying associated genes involved in specification and differentiation of cell types among both cell populations.

**Fig. 6:**
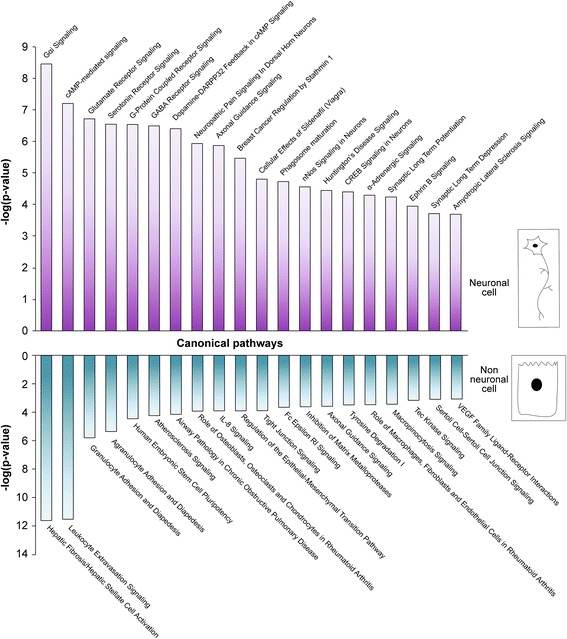
Pathway analysis of the differentially expressed genes in the neuronal and non-neuronal cells of the zebrafish intestine. Pathway analysis of the upregulated, downregulated, and the complete list of significantly differentially expressed genes was done using IPA. The *purple bar graph* represent the top 20 canonical pathways predicted to be most likely to be activated in the neuronal genes using the genes most significantly enriched in the neuronal population while the *blue bar graph* represents the top 20 canonical pathways predicted to be most likely to be activated in the neuronal genes using the genes most significantly downregulated in the non-neuronal population

To obtain more information on signaling pathways that can be associated with our datasets, and to gain insight into the genes comprising these pathways, additional analysis was carried out. IPA pathway analysis was used to determine the molecular pathways that were highly represented in either the neuronal or non-neuronal dataset. The final analysis was done using 1133 enriched genes and 696 of depleted genes of the DEG genes between the neuronal and non-neuronal populations. Figure 6 shows the top 20 enriched canonical pathways in the enhanced as well as the depleted list of genes. The top 10 canonical pathways along with their –log(*p*-values) and associated genes are shown in Table 2. The complete information about the pathways can be found in Additional file 23: Table S4. As expected, ‘G-protein coupled receptor-signaling’ pathway, ‘cAMP-mediated signaling’, ‘nNOS signaling’ pathway and ‘axon guidance’ were among the highly enriched pathways in the neuronal population (Fig. 6). These pathway predictions implicated many additional genes as up-regulated, and, consistently, the transcript levels within our neuronal transcriptome confirmed these expectations (data not shown). Conversely, some of the significant pathways in the depleted list comprised of ‘IL-8 signaling pathway’, ‘Tec kinase signaling’, and ‘inhibition of matrix metalloprotease’. The first two pathways are known to regulate the immune system [81–83]. Interestingly, the third identified pathway involving matrix metalloproteases is known to induce cell migration [84]. Thus, the enrichment of inhibition of this pathway may suggest a mechanism for cessation of cell migration. This potential function would be consistent with this developmental stage in which a proportion of enteric neural crest cells has completed the migratory phase, has differentiated and is functionally capable of driving intestinal motility.

**Table 2:** Top 10 canonical pathway

Canonical pathways	Negative log(*p*-value)	Molecules
Gαi Signaling	8.462	GRM2,GABBR1,CNR1,GRM3,DRD2,GRM4,GNG3,ADCY8,S1PR1,OPRK1,RGS7,HTR1D,ADCY7,ADRA2B,OPRL1,GNB2,GNB5,RGS14,MAPK1,ADORA3,GNG2,ADRA2C,CHRM4,ADRA2A,HRAS
cAMP-mediated signaling	7.209	GRM2,PDE4A,GNAO1,GABBR1,CNR1,GRM3,DRD2,GRM4,HTR7,PDE4D,HTR5A,ADCY8,AKAP6,DUSP4,S1PR1,OPRK1,HTR1D,RGS7,ADCY7,CAMK4,ADRA2B,CAMK1D,PPP3CA,OPRL1,PTGER2,RGS14,MAPK1,ADORA3,CAMK1G,ADRA2C,CHRM4,ADRA2A,PDE1A
Glutamate Receptor Signaling	6.722	GRM2,GRIA1,GRID1,CAMK4,GRIN2B,GRM3,GRIN3B,SLC1A7,DLG4,GRM4,GRIK4,GRID2,SLC1A6,GNG2,GRIK3
Serotonin Receptor Signaling	6.551	HTR1D,ADCY7,DDC,GCH1,HTR2B,TPH1,HTR7,HTR3B,SLC18A1,SLC18A2,HTR5A,SLC18A3,ADCY8
G-Protein Coupled Receptor Signaling	6.542	GRM2,PDE4A,GNAO1,GABBR1,CNR1,HRH1,GRM3,GNAQ,DRD2,GRM4,HTR7,PDE4D,HTR5A,ADCY8,DUSP4,S1PR1,PRKCE,OPRK1,HTR1D,RGS7,ADCY7,CAMK4,ADRA2B,SYNGAP1,OPRL1,PTGER2,HTR2B,RGS14,MAPK1,ADORA3,ADRA2C,CHRM4,ADRA2A,HRAS,PDE1A
GABA Receptor Signaling	6.499	GABRG2,ADCY7,GABBR1,GPR37,GABRB2,DNM1,NSF,GABRB3,KCNH2,KCNN3,GABRA5,GABRA1,SLC6A1,GABRB1,KCNQ3,ADCY8
Dopamine-DARPP32 Feedback in cAMP Signaling	6.41	KCNJ2,PRKG2,KCNJ6,GRIN3B,KCNJ9,GNAQ,DRD2,NOS1,PPP1R14B,PRKCZ,ADCY8,CSNK1E,PRKCE,PPP2R5B,ADCY7,PRKG1,CAMK4,KCNJ14,PPP2R2C,PPP3CA,CACNA1A,GRIN2B,PPP1R14C,KCNJ3,CACNA1C,PRKD1
Neuropathic Pain Signaling In Dorsal Horn Neurons	5.939	PRKCE,GRM2,TACR1,GRIA1,GPR37,CAMK4,CAMK1D,GRIN2B,GRM3,KCNH2,GRIN3B,GRM4,KCNN3,MAPK1,PRKD1,NTRK2,CAMK1G,PRKCZ,KCNQ3
Axonal Guidance Signaling	5.875	SEMA4D,LINGO1,SRGAP3,GNAO1,SLIT3,SEMA6D,DPYSL5,RGS3,PAK7,TUBB2A,GNAQ,EPHA6,TUBA1A,ARHGEF7,GNG3,ADAM11,RAC3,KLC1,NTRK3,FZD3,NTRK2,PRKCZ,UNC5D,EFNA2,PLXNA3,PRKCE,TUBB,ADAM8,ADAM23,ADAM19,PPP3CA,EPHA7,RTN4R,GNB2,TUBB4A,ADAM22,SLIT2,NGFR,GNB5,PAK6,SHANK2,TUBA1B,MAPK1,NTRK1,PRKD1,GNG2,HRAS,L1CAM
Breast Cancer Regulation by Stathmin1	5.472	TUBB2A,GNAQ,TUBA1A,ARHGEF7,PPP1R14B,GNG3,PRKCZ,ADCY8,PRKCE,PPP2R5B,TUBB,ADCY7,CAMK4,CAMK1D,PPP2R2C,STMN1,GNB2,TUBB4A,PPP1R14C,GNB5,TUBA1B,MAPK1,PRKD1,CAMK1G,GNG2,ARHGEF9,HRAS

### Differentially expressed genes may drive development and differentiation of the ENS

In examining our transcriptomic data, we noted many genes to be highly enriched within the neuronal population compared to the non-neuronal population. These genes could be further divided in distinct categories according to their possible function as candidates for ENS development and differentiation (Table 3). Examples of molecular categories represented in the data were DNA and RNA binding proteins, signaling molecules, neuropeptides, and proteins involved in synaptogenesis. Interestingly but not surprising there is a significant enrichment in several neuropeptide genes such as *Vasoactive intestinal peptide (vip)* [43] and *secretogranin II (scg2b)*, both of which are implicated in ENS function. The cytokine gene, *secretogranin II (scg2)*, from which the neuropeptide secretoneurin is derived, exhibits the highest enriched fold change in expression in the neuronal population. Though, Secretoneurin is expressed in rat and human myenteric and submucosal ganglia [85, 86] and has been implicated in the regulation of motility of the gastrointestinal tract [86, 87], no study involving loss of function in the ENS has been reported [88].

**Table 3:** Potential candiate genes with their log2foldchange

Gene Ontology Category	Gene	log2foldchange
DNA binding protein	phox2bb^a^	8.1973
	phox2a^a^	8.0614
	tlx2	4.9425
	**chd5**	3.8181
	**isl2a**	2.4776
RNA binding protein	elavl3^a^	6.9122
	elavl4^a^	5.8957
Neuropeptide	vip^a^	8.7919
	scg2b	8.06145
	nmu	7.4064
	**cart2**	7.2399
	vip2	6.5241
Signaling molecules	**fgf13b**	5.9157
	**chn1**	3.1659
	ret^a^	3.7052
	**dkk1b**	3.3082
	fgf13a	2.9688
Synaptic	syn2a	4.9865
	syt1a	5.3126
	Stx1b	5.0042
	Stxbp1a	4.3685
	Stxbp1b	6.17369
	Snap25b	5.6228
	Snap25a	4.8107
	cplx2	4.3900

^a^Previously described genes in the enteric nervous system

Potential novel candidate genes are bolded

Genes such as *synapsin II,* (*syn2a)* and *snap25*, required for synaptic vesicle formation and fusion and neurotransmitter release [89], are similarly enriched, consistent with the establishment of synapses and a functioning neural network. The transcription factor *tlx2*, required for ENS development and function [90–92], is enriched, as is *Islet 2a (isl2a),* a transcription factor known to play a role in motoneuron specification in the CNS [93]. The newly identified ENS related genes can be subdivided into three main categories: (1) genes known to be expressed by neurons outside the ENS but not yet associated with ENS development; (2) genes reported to be expressed in the ENS but for which functional studies have not yet been reported; and (3) novel genes that have not yet been associated with expression or function during ENS development. A more detailed discussion of Table 3 follows below. Thus, our approach successfully identified genes and pathways known to be involved in neurogenesis and synaptogenesis, functions that are expected to be present in the ENS cell population. Furthermore, this suggests also that our dataset uncovers reliably novel genes or pathways that have not been implicated in ENS development yet, and can be used as a comprehensive resource to start further investigations into their roles in ENS development and differentiation.

## Discussion

The enteric NCCs are a multipotent cell population that originates in the neural tube and migrates throughout the embryo, proliferates and eventually differentiates to give rise to the neurons and glia of the enteric nervous system [94]. The development of ENS is complex directed by a number of cellular and molecular processes. To better understand the molecular mechanisms of enteric crest differentiation from precursors to a mature, fully functional ENS, we generated a transcriptomic profile of the zebrafish ENS at 7dpf. By 7dpf, the neural crest cells have already migrated into and populated the larval zebrafish intestine. At this stage in development, enteric NCCs have differentiated or are in the process of differentiating into the neurons and glia of the ENS [49, 51]. The composition of the ENS is likely to be a combination of fully differentiated enteric neurons and glia as well as enteric neural crest cells undergoing proliferation and differentiation. Using a transgenic line marking the phox2b expressing neural crest cells we aimed to build a lineage-specific transcriptional profile of progenitors and progenitor derivatives of the ENS.

Previous microarray experiments comparing animals with and without a complete ENS or cultured neurospheres, and RNA-seq expression analysis on culture-propagated human embryonic stem cells, or anterior intestinal tracts have revealed a number of genes important for ENS transcriptome development [1, 95–97]. However, here we expand these previous attempts to obtain a more comprehensive unbiased analysis of the enteric transcriptome. Our dataset includes low abundance and variable transcripts expressed in the ENS along the entire intestine during normal development in zebrafish. This expands our knowledge of the vertebrate ENS in itself, and opens up molecular comparisons between the ENS in higher and lower vertebrates by providing in depth gene expression profiles of enteric neural crest and their derivatives in this species. To complement this analysis we also generated a transcriptomic profile of the cells surrounding the ENS, the cellular microenvironment comprised by the endo- and mesodermally-derived gut tissue. Importantly, to minimize the need to amplify the RNA prior to sequencing and potential amplification bias prior to sequencing we dissected and FAC sorted over 4700 larval intestines. Our initial analyses suggest that this strategy allowed us to accurately and reproducibly determine transcript abundance over a wide dynamic range throughout the genome.

Our experimental strategy included using the zebrafish *Tg(phox2b:EGFP)* ^*w37*^ line to mark GFP-positive ENS cells, the manual dissection of the intestine to separate GFP-positive ENS cells from other GFP-positive cells, the physical dissociation and FAC sorting of the intestinal cells into the GFP-positive ENS/neuronal cells, and GFP-negative non-neuronal cells, RNA-sequencing of samples with two biological replicates, and bioinformatics procedures to map reads to the zebrafish genome, and determine read counts for individual genes genome-wide. The success of our experimental strategy was validated by demonstrating that (1) ectopic GFP and endogenous *phox2b* transcripts almost exclusively enriched in the GFP-positive cell population, (2) housekeeping genes were similarly expressed in both cell populations, and (3) RNA-seq and qPCR results consistently showed a strong enrichment of candidate neuronal genes (*phoxb*, etc) and candidate non-neuronal genes (*amy2, fli-1*) in the expected cell populations, respectively. Furthermore, subsequent bioinformatics analyses demonstrated that expression of genes with GO terms associated with ‘neuronal’ features were enriched in the GFP-positive cell population, and genes with GO terms associated with ‘non-neuronal’ features were enriched in the GFP-negative cell population (discussed in more detail below). Thus, our datasets yielded meaningful transcriptional profiles to identify the abundance of specific ENS and non-neuronal intestinal genes genome-wide.

### The GFP-positive neuronal cell population: Profiles of the ENS

Taking an RNA-seq approach, we isolated the enteric neurons from the zebrafish intestine and compared gene expression profiles between the neuronal and non-neuronal populations by carrying out a differential gene expression analysis. We identified 2561 genes (Additional file 2) that showed enrichment in the transcriptome of the enteric neuronal population as compared to the non-neuronal population of cells. These represent a diverse set of molecular and cellular genes and pathways that were further examined by various ‘pathway’ analyses.

The top 20 pathways showing the most significant enrichment activity in the neuronal population included ‘G protein-coupled receptor signaling’, ‘axon guidance’, and several well-studied neurotransmitter signaling pathways. G protein-coupled receptors have been found to be very important in neurogenesis and anomalies in members of this superfamily are known to cause aganglionosis in the intestine of mammals [98–100]. Other pathways that showed high activity in the enteric population included many of the well-studied neurotransmitter signaling pathways, the serotonin receptor, GABA receptor and NOS signaling. Serotonin receptors have been associated with abnormal motility of the gastrointestinal tract of mammals [101–103], and also associated with such diseases as inflammatory bowel disease and irritable bowel syndrome [104].

To find the specific functional, molecular and structural gene categories, we performed a GO enrichment analysis. The GO enrichment pathway analysis can be viewed in a hierarchy of GO terms where several terms are nested under other terms. The complete analysis gave rise to an extended hierarchy that consisted of hundreds of nodes, for example, the biological process hierarchy of the depleted list consisted of 280 nodes and 467 edges. Subsequent manual editing created a second core hierarchy reducing unnecessary complexity for easier visualization. Expectedly, our analysis revealed terms such as ‘neurogenesis’, and ‘axonogenesis’, that were enriched within the GFP-positive neuronal population. We also observed an enrichment of molecules characteristic of neurons including signal transduction, metal ion transport, neuron differentiation and cell adhesion. For example, genes such as ROBO4, SLIT2, NADL1.1, PLXN, etc. (see Additional file 2) were identified that play key roles in cell migration and axon guidance during nervous system development.

While there were common terms in both analyzed intestinal cell populations, the significance level showed clear differentiation between the two groups. For example, the term nervous system development has a *p*-value of 2.62E-10 in the hierarchy specific to the neuronal population while has *p*-value lower than <5.00E-10 in the non-neuronal GO enrichment hierarchy. Thus, the term ‘nervous system development’ is much more significant in the neuronal population as compared to the non-neuronal population consistent with a higher enrichment of genes associated with neurogenesis and neuron development in the GFP-positive population. As expected, similar terms associated with neuron differentiation, axonogenesis, neurogenesis, etc. are also found enriched in this hierarchy.

When a subset of the GO terms was grouped according to molecule type (Fig. 5) further distinct differences could be seen. The neuronal population had an overall higher number of different types of transporters and ion-channels. Ion-channel genes were the most significant increase in molecule type in the neuronal population. 8% of the genes that fell into the ‘ion-channel’ category, were genes associated with voltage-dependent calcium, potassium or sodium channels reflective of their role in generating and modulating neural activity.

### Axon guidance molecules in the ENS

As enteric neuron precursors are differentiating and forming neural networks within the intestine at this stage in development it is not surprising that ‘axonal guidance signaling’, was also enriched in the neuronal dataset. Among the axon guidance molecules indicated as enriched by the pathway analysis were the Sema, Plxn, Nadl1, Fxd3 and Slit genes.

The Semaphorin (sema) family’s role in axon guidance is well documented [105] but recent evidence demonstrates that semaphorins play also an important role in ENS development [106–109]. In zebrafish the knockdown of Sema3C and Sema3D results in the loss of enteric neurons in a dose dependent manner [109–111]. GWAS studies have also identified polymorphisms at the Sema3C and Sema3D loci as possible at-risk alleles for HSCR [109].

Interestingly, our analysis suggests a novel role for the Nogo/Reticulon pathway in ENS development. The Nogo/Reticulon pathway has been implicated in the inhibition of axon growth cones and myelination [112]. Our data demonstrates that Reticulon4 receptor (RTN4R), also known as the Nogo receptor, is upregulated in the ENS population. Concomitantly, leucine-rich repeat Ig domain-containing Nogo-interacting protein 1 (LINGO1) known to mediate the collapse of growth cones in the presence of certain myelin proteins [113] is also upregulated in our data. RTNR4 and LINGO1 form a complex with the p75neurotrophin receptor to mediate the inhibition of growth cones [114]. Although a role for this axon guidance pathway has not been implicated in ENS development, reticulon4 (RTN4/Nogo) is expressed in the mammalian adult ENS [115]. It will be exciting to see whether further studies can confirm definitive roles for LINGO1, and RTNK4 in ENS development.

In addition to the axonal guidance molecules, other gene families found within the neuronal dataset, the ADAM and neurotrophic tyrosine receptor kinase (NTRK) families are known to have a role in axon guidance. A disintegrin and metalloprotease (ADAM) is from the Metzincins superfamily of metalloproteases [116]. This family has been shown to play a very important role in development by regulating cell migration, differentiation, cell-cell interaction, and receptor-ligand signaling [117, 118]. Interestingly, some of the axonal guidance pathway genes enhanced in our dataset have been implicated in the development of the nervous system. For example, ADAM22 is necessary in PNS development and deficiency leads to hypomyelination of peripheral nerves and ataxia [119]. ADAM22 deficient mice display defects in the proliferation and differentiation of glia [120]. The neurotrophic tyrosine receptor kinase (NTRK) family comprises of receptors that are required to maintain synaptic strength and plasticity in the nervous system [121]. The three genes associated with axonal guidance from this family are NTRK1, NTRK2 and NTRK3. Similar to the ADAM family, all of these genes are highly expressed in the neuronal population in our dataset.

From our analyses we have identified both genes known to be expressed in and required for enteric neuron development, as well as candidate genes that may play a role in, enteric neuron differentiation, enteric axon guidance and connectivity. Among these genes, we found some known to be required for ENS development in other model organisms; others known to be required for neuronal development in the CNS but not directly tested for a role in ENS development; and finally genes not known to play a role in either neuronal development either in the CNS or ENS. Based on expression levels in our dataset, the latter two categories of genes may prove to be interesting candidates for further investigation. To illustrate these categories, we selected a few genes, with different molecular functions, from our enriched dataset and searched the literature for what was known about their role in ENS or neuronal development in general (Table 3).

### Neuropeptides in the ENS cell population

Not unexpectedly, the neuropeptide class of molecules was also significantly enriched in the GFP-positive population. Vasoactive intestinal peptide (Vip), NeuromedinU (Nmu), Cocaine and amphetamine-regulated transcript2 (Cart2), Secretograinin II (Scg2b) and Neuromedin b (Nmba) are neuropeptide precursors, neuropeptides, or members of neuropeptide families, expressed during and required for ENS development and function [122–127].

Sectretoneurin, a functional neuropeptide expressed in the ENS, is derived from the secretograinin II gene [85, 86]. The CART genes have been associated with appetite and energy control in zebrafish [128], and with energy balance in chicken [129]. Cart is expressed in a subset of Ret-positive enteric neurons in the murine intestine, and is reported to be one of the “earliest markers” of the ENS [1]. In their study Heanue and Pachnis also showed that Cart positive neurons represented a subset of enteric neurons [1, 130, 131]. Duplication events in the zebrafish genome have resulted in 4 cart genes compared to a single gene, Cart propeptide (Cartpt), in mouse [132, 133]. We find that in our dataset only Cart2 seems to be enriched (Additional file 2: Table S1). Together the expression of cart neuropetides in our data and these other studies suggest the CART family of nueopepetides may play important roles in ENS development and/or function. A more definitive role for the CART family of neuropetides in ENS function await further studies.

### Enrichment of synaptogenesis genes in the ENS

Synapse assembly and disassembly is an important part of the formation and maintenance of neural circuitry in the developing nervous system as well as plasticity in the mature nervous system [134, 135]. As expected we found many components in this category to be enriched in our dataset. Interestingly, our transcriptomic data may also show evidence that genes whose protein products form functional complexes together may have similar levels of enrichment. An example of this is proteins aligned with synaptogenesis and synaptic vesicle fusion (Table 3). Syntaxin 1 (Stx1), Syntaxin binding protein 1 (Stxbp1) and SNAP-25 are all components of the SNARE [(soluble NSF attachment protein) receptor] complex of presynaptic proteins that facilitate the fusion of synaptic vesicles with the plasma membrane during the release of neurotransmitters [89]. Inhibitors of SNARE proteins have been shown to decrease enteric neural crest migration and ENS precursor neurite extension [136].

 Complexin II (Cplx2), Synapsin II (Syn2), and Synaptotagmin 1 (Syt1) modulate vesicle fusion and neurotransmitter release. Syt1 is a synaptic vesicle transmembrane protein involved in Ca^2+^-sensitive triggered neurotransmitter release expressed in enteric neurons [89, 136, 137]. Syn2 a neuron-specific phosphoprotein, assists in clustering and recycling of synaptic vesicles [138]. Cplx2 is a soluble pre-synaptic protein that binds the SNARE complex aids in the fusion of synaptic vesicle but negatively regulates vesicle fusion [137, 139]. In mammals the two paralogous genes *stx1A* and *stx1B* are often expressed in the same cells, and function often redundantly [140]. Both Syntaxin 1A and 1B are expressed in the murine myenteric plexus but it is not clear whether they are functionally redundant as they localize to different regions of the neuron [141]. Snap-25 is also expressed in the ENS of rodents and humans and localizes to the presynaptic membrane of cholinergic and nitrergic enteric neurons [136, 142–144]. During vertebrate evolution, the teleost fish lineage experienced an additional genome duplication [145, 146]. As with other genes this led to duplications and retention of additional *stxbp1* and the *snap-25* genes in the zebrafish genome. Thus, the zebrafish genome contains two *stxbp1* paralogues, *stxbp1a* and *1b* and two paralogues of *snap-25, snap25a* and *2b*. Currently, it is not known which of these paralogues are required for vesicle docking and fusion in the zebrafish enteric neurons, however our data shows that all the major protein components of the presynaptic vesicle fusion are significantly enriched in our neuronal transcriptomic profile. Interestingly, these genes appear to be enriched at similar levels, between 4.36 and 6.17 with an average log2fold change expression of 5.083 (see Table 3) including *stxbp1a* and *snap 25*A but not s*txbp1b* and *snap 25B*. It is tempting to speculate that the observed expression levels may predict the specific paralogue contributions to a protein complex such as the vesicle fusion machinery in the ENS cells.

### The DNA-and RNA binding proteins of the ENS

As transcription factors are generally required to drive the specification and differentiation of developmental processes we initially surveyed genes that have been described to be expressed in enteric neural crest, or enteric neurons. Our cell selection process was based on cells presumed to express *phox2b* (see above), so we examined the gene expression levels of *phox2b* and its paralogue *phox2a*. Both *phox2a* and *phox2b* were expressed at log2foldchange higher than 4. *phox2b* is known to drive the specification and differentiation of enteric neural crest and is required for the normal development of a complete and functional ENS [53–55]. Phox2a is also similar to Phox2b in its expression in the enteric neurons, cranial glia and other differentiating autonomic neurons [64, 147–149] but its expression appears to be more restricted to a subset of enteric neural crest [150]. Both Phox2b and Phox2a have been shown to transcriptional regulate Tlx2 [91]. Loss of Phox2b gives rise to aganglionic gastrointestinal tract [54] while in the absence of Phox2a some enteric neurons are present in the oesophogeal region [151]. Whether more caudal enteric neurons are present or whether more subtle phenotypes such as the loss of specific subtypes of enteric neurons can be found in Phox2a deficient mice is not clear.

Further transcription factors that are enriched in the ENS cell population included T-cell leukemia homeobox 2 (Tlx2), Islet2a (Isl2), and Chromodomain helicase DNA binding protein 5 (Chd5). Interestingly, Tlx2 (Hox11l) has been shown to be a target for Phox2b [152] and Phox2a [90, 91]. Tlx2 deficiency leads to megacolon and myenteric neuronal hyperplasia in mice [90–92], which may explain its association with intestinal motility disorders like Intestinal neuronal dysplasia (IND). Isl2a, a LIM homeobox transcription factor, plays a critical role in lineage specific differentiation of motoneurons in the spinal cord [93, 153].

Chd5 encodes a chromatin remodeling protein that is specific to neurons [154] and promotes neurogenesis by activating the transcription of neural differentiation genes and inhibiting genes for non-neural fates [154–158]. Isl2a and Chd5 are required for neuronal differentiation in the CNS, but have not been implicated in ENS development, and may represent novel candidates required for ENS specification or differentiation.

At day 7 of larval development, some proportion of neural crest cells are differentiated and forming a functioning neuronal network within the ENS. Consistently we found, *elavl3* and *elavl4* two pan-neuronal RNA binding proteins expressed in postmitotic neurons were equally highly enriched with log2foldchanges of 6.9122 and 5.8957 respectively. This significant enrichment is consistent with increasing number of differentiated enteric neurons [159–163] at this time point in zebrafish development.

### Signaling molecules within the ENS

An additional category of genes we selected to sample was signaling molecules. Ret tyrosine kinase receptor was significantly enriched in our neuronal population with a log2folchange of 3.7, consistent with its role in ENS development (see earlier discussion). Fibroblast growth factor 13 (Fgf13) is a members of the Fibroblast growth factor (FGF) family [164]. Although Fgf13 shares structural similarities with the FGF family, it operates as an intracellular FGF that is neither secreted from the cell as other FGFs, nor does it activate the canonical FGF signaling pathway through FGF tyrosine kinase FGF receptors. Fgf13 is highly expressed in the embryonic CNS and has been shown to regulate neural development by stabilizing microtubules [165–167]. Mice deficient in FGF13 have defects in axonal branching, learning and memory. In addition to the CNS, Fgf13 expression is also present in the enteric ganglia of mice [136, 168]. In zebrafish there are two Fgf13 genes, *fibroblast growth factors 13a and 13b (fgf13a and fgf13b*, respectively) [169]. *fgf13b* is expressed in the developing zebrafish ENS, and although not yet examined in the ENS, *fgf13a* is expressed in neurons of the CNS [170, 171]. Our data demonstrates that both *fgf13a* and *fgf13b* are expressed in transcriptome of the developing ENS, suggesting FGF13 signaling may play a role in enteric nervous system development or function.

Among the candidate genes identified from our transcriptomic profiling that could play a signaling role in ENS development and/or function is Chimerin1 (Chn1). Chn1 a GTPase-activating protein expressed in neurons in embryogenesis during a time of differentiation and synaptogenesis [172]. To our knowledge, the expression of, or a functional role for Chn1 in ENS development has not been investigated. Intriguingly, a search of Mouse Genome Informatics (MGI) database and the International Mouse Phenotyping Consortium (IMPC) identified a transgenic reporter line, Chn1^tm1.1(KOMP)Vlcg^, driving LacZ expression under the Chn1 promoter. LacZ positive cells can be seen in the gastrointestinal tract in locations consistent with the position of the myenteric plexus [173–175] suggesting a potentially conserved role of chn1 in vertebrate ENS development.

Dickkopf 1 (Dkk1) belongs to the Dickkopf family of sectreted proteins that act as inhibitors of the Wnt-signaling pathway. Wnt signaling can induce synaptic protein clustering, promote the assembly development and maintenance of synapses [176–178]. Dkk1 inhibits Wnt causing the disassembly of synapses in mature neurons [176, 179]. It may be that an enrichment of Dkk1 expression in the enteric neuronal population data reflects a role for Dkk1 in synapse disassembly, an essential part of the wiring and refinement process within the developing ENS neural circuitry (Table 3) [134].

### The GFP-negative non-neuronal cell population of the intestine

Although the goal in this study was to define the ENS (GFP positive) transcriptome, the additional transcriptome of the sorted GFP-negative non-neuronal cell population of the intestine serves not only as an important control for our experimental strategy (see above), but it provides a powerful complementary data set in itself. The non-neuronal tissue of the intestine is independently formed during development. Endodermal and mesodermal tissues form the digestive, vasculature, and muscle derivatives of the intestine, respectively. Thus, the GFP-negative cell population should be defined by entire different transcriptional signature.

Indeed, our GO term analysis found the expected enrichments for terms like proteolysis, blood vessel development and vasculature development to be very specific to the GFP-negative cell population of the intestine. By contrast, the term nervous system development had a much lower *p*-value (<5.00E-10) in the non-neuronal GO enrichment hierarchy than in the ENS cell population. Similarly, while the neuronal population had a higher overall number of different types of transporters and ion-channels, the non-neuronal cells had a higher number of transcriptional regulators and peptidase genes. We also observed high expression of ‘peptidases’ genes chymotrypsinogen B2, chymotrypsin-like, and several members of the carboxypeptidase, protease and matrix metallopeptidases families that reflect, molecularly define, and are consistent with the digestive nature of the large majority of the GFP-negative cell population. Interestingly, of the few ion channels expressed in the non-neuronal population, the cystic fibrosis transmembrane conductance regulator (CFTR) channel was significantly enriched. CFTR is expressed in the intestinal epithelium and is required to maintain water homeostasis in the intestine [180]. The differential appearance of this gene in the non-neuronal population suggests our GFP-negative cell profile represents the intestinal microenvironment.

Among the pathways enriched in the GFP-negative non-neuronal population pathways, and downregulated in the neurons, are genes associated with the vasculature and tight junction formation and regulation (see Additional file 23: Table S4). The regulation of the ‘epithelial to mesenchymal transition’ and ‘axon guidance’ pathways were also downregulated in the neuronal population. The latter initially may seem counter intuitive, however, an examination of the genes within the ‘downregulated’ axon guidance category revealed genes like Shh and BMP, growth factors expressed in the endoderm and mesoderm of the developing intestinal tract and known to affect enteric neural crest migration and proliferation (see Additional file 23: Table S4) [181–189].

As indicated above the independently derived non-neuronal intestine provides a substrate for the arriving neuroectodermally derived ENS cell population. This cellular microenvironment must provide signals to guide migration, proliferation, penetration, settlement, and differentiation of the ENS cells along the entire intestine in a non-cell autonomous manner to ensure the full functional innervation along the entire developing gut. This process must be dependent on multiple interactions between these cell populations, and our data sets should have captured this molecular dialogue at a particular important and sensitive developmental time when this connection is being established. Thus, closer examination of this GFP-negative non-neuronal data set may provide a starting point to molecular dissect these little known interactions on a molecular level.

## Conclusion

We report a comprehensive gene expression profile of a population of enteric neural crest progenitors and their enteric nervous system derivatives during normal development. We also report the gene expression profile of the cells that constitute the microenvironment that the enteric crest and derivative reside in, the intestinal tract. Our results confirm previously reported genes and pathways known to be required for ENS development and function, as well as novel genes and pathways, suggesting that this dataset provides valuable new insights into the genetic, cellular and molecular mechanisms driving the development and maintenance of a functioning ENS.

## Additional files

Additional file 1: Figure S1. Fluorescent Activated Cell Sorting (FACS) of the dissociated zebrafish. Fluorescent Activated Cell (FAC) analysis of sorted GFP-positive and GFP-negative cells. Viable cells separated into GFP-positive cells (red dots) and GFP-negative cells (blue dots). The table below gives an approximate survival and sorting rate of the sample. This particular analysis had 44.3% of live cells, among which 3.6% were positive and 96.4% were negative. (TIFF 1.48 mb)

Additional file 2: Table S1. List of differentially expressed genes between the two cell populations of the intestine. The list in the first sheet represents all the genes filtered based on their adjusted *p*-value (cutoff is 0.01) while the second sheet shows the genes further filtered based on log2foldchange (1.5). Sheets 3 and 4 show the separated gene lists from the second sheet into upregulated genes in the neuronal cells and downregulated genes in the neuronal cell population, respectively. The scatter plot and the heat map in the main paper (Fig. 3c and d) were generated using the data provided in this table. (XLSX 3109 kb)

Additional file 3: Figure S2. The complete hierarchy showing the Biological processes category of GO enrichment in neurons. GO enrichment analysis of biological processes was performed with 530 genes which resulted in 143 enriched GO terms (nodes) and 227 edges. The size of the nodes refers to the number of genes associated with the GO term while the color represents the significance of association with the GO term. The low significant nodes to the higher significance ranges from white to dark orange nodes. (PNG 1.41 mb)

Additional file 4: Figure S3. Top 50 significant GO terms from the biological process category in the neurons. A bar graph illustrating the first significant 50 biological processes GO term nodes based on the *p*-value. The y-axis shows the top 50 GO terms for the biological processes, while the x-axis shows the corresponding –log(*p*-value). The specific values can be found in the additional file 21. (JPG 82 kb)

Additional file 5: Figure S4. Top 50 GO categories based on the highest percentages of genes associated with the GO terms. A bar graph illustrating the first 50 nodes based on the percentage of genes associated with a specific GO term. The y-axis shows the top 50 GO terms for the biological processes, while the x-axis shows the corresponding percentage of genes. The specific values can be found in the Additional file 21. (JPG 93.2 kb)

Additional file 6: Figure S5. The complete hierarchy showing the Biological processes category of GO enrichment in non-neurons. A GO enrichment analysis of biological processes in the non-neuronal genes. The analysis was performed with 482 genes which resulted in 280 enriched GO terms (nodes) and 467 edges. The size of the nodes refers to the number of genes associated with the GO term while the color represents the significance of association with the GO term. The low significant nodes to the higher significance ranges from white to dark orange nodes. (PNG 1.14 mb)

Additional file 7: Figure S6. Top 50 significant GO terms from the biological process category in the non-neurons. A bar graph plotted with the top 50 significant GO terms nodes based on the *p*-value. The y-axis shows the top 50 GO terms for the biological processes, while the x-axis shows the corresponding –log(*p*-value). The specific values can be found in the additional file 21. (JPG 97.7 kb)

Additional file 8: Figure S7. Top 50 GO categories with the higher percentage of genes associated with a specific GO term in the non-neurons. A bar graph plotted with the first 50 nodes representing the highest percentage of genes associated with a specific GO term. The y-axis shows the top 50 GO terms for the biological processes, while the x-axis shows the corresponding percentage of genes. The specific values can be found in the additional file 21. (JPG 105 kb)

Additional file 9: Figure S8. The complete hierarchy showing the Molecular function category of GO enrichment in neurons. A GO enrichment analysis of specific molecular function the neuronal genes. The analysis was performed with 530 genes which resulted in 114 enriched GO terms (nodes) and 146 edges. The size of the nodes refers to the number of genes associated with the GO term while the color represents the significance of association with the GO term. The low significant nodes to the higher significance ranges from white to dark orange nodes. (PNG 1.26 mb)

Additional file 10: Figure S9. Top 50 significant GO terms from the Molecular function category in neurons. A bar graph was plotted with the first 50 significant GO term nodes based on the *p*-value. The y-axis shows the top 50 GO terms for the molecular function, while the x-axis shows the corresponding –log(*p*-value). The specific values can be found in the additional file 21. (JPG 111 kb)

Additional file 11: Figure S10. Top 50 GO categories with the highest percentage of neuron associated genes. A bar graph plotted with the first 50 nodes based on the percentage of genes associated with a particular GO term. The y-axis shows the top 50 GO terms for the molecular function, while the x-axis shows the corresponding percentage of genes. The specific values can be found in the additional file 21. (JPG 106 kb)

Additional file 12: Figure S11. The complete hierarchy showing the Cellular component category of GO enrichment in neurons. A graph of the cellular component GO term analysis of the neuronal genes. The analysis was performed with 530 genes which resulted in 75 enriched GO terms (nodes) and 126 edges. The size of the nodes refers to the number of genes associated with the GO term while the color represents the significance of association with the GO term. The low significant nodes to the higher significance ranges from white to dark orange nodes. (PNG 824 kb)

Additional file 13: Figure S12. Top 50 significant GO terms from the Cellular component category in neurons. A bar graph with the top 50 most significant nodes based on the *p*-value. The y-axis shows the top 50 GO terms for the cellular component, while the x-axis shows the corresponding –log(*p*-value). The specific values can be found in the additional file 21. (JPG 73 kb)

Additional file 14: Figure S13. Top GO categories with the largest number of gene association in neurons. A bar graph was plotted with the top nodes based on the percentage of genes associated with a particular GO term. The y-axis shows the top 41 GO terms for the molecular function, while the x-axis shows the corresponding percentage of genes. The small percentage of gene associated with GO terms beyond the 41st category were too low to be plotted. The specific values for the 41 nodes can be found in the additional file 21. (JPG 65 kb)

Additional file 15: Figure S14. The complete hierarchy showing the Molecular function category of GO enrichment in non-neurons. A GO enrichment analysis of molecular function in the non-neuronal genes. The analysis was performed with 482 genes which resulted in 81 enriched GO terms (nodes) and 92 edges. The size of the nodes refers to the number of genes associated with the GO term while the color represents the significance of association with the GO term. The low significant nodes to the higher significance ranges from white to dark orange nodes. (PNG 1.22 mb)

Additional file 16: Figure S15. Top 50 significant GO terms from the Molecular function category in the non-neurons. A bar graph was plotted with the top 50 significant GO term nodes based on the *p*-value. The y-axis shows the top 50 GO terms for the molecular function, while the x-axis shows the corresponding –log(*p*-value). The specific values can be found in the additional file 21. (JPG 97.3 kb)

Additional file 17: Figure S16. Top 50 GO categories with the largest number of gene associated with GO terms in the non-neurons. A bar graph plotted with the top 50 nodes representing the highest percentage of genes associated with a specific GO term based on the percentage of genes associated. The y-axis shows the top 50 GO terms for the molecular function, while the x-axis shows the corresponding percentage of genes. The specific values can be found in the additional file 21. (JPG 88.5 kb)

Additional file 18: Figure S17. The complete hierarchy showing the Cellular component category of GO enrichment in non-neurons. A GO enrichment analysis of cellular function in the non-neuronal genes. The analysis was performed with 482 genes which resulted in 42 enriched GO terms (nodes) and 61 edges. The size of the nodes refers to the number of genes associated with the GO term while the color represents the significance of association with the GO term. The low significant nodes to the higher significance ranges from white to dark orange nodes. (PNG 714 kb)

Additional file 19: Figure S18. Top significant GO terms from the Cellular component category in non-neurons. To identify and visualize some of the top significant GO terms, a bar graph was plotted with the top nodes based on the *p*-value. The y-axis shows the top 25 GO terms for the cellular component, while the x-axis shows the corresponding –log(*p*-value). The nodes with significant *p*-values beyond the 25th category were too low to be plotted. The specific values for the 25 nodes can be found in the additional file 21. (JPG 63.7 kb)

Additional file 20: Figure S19. Top GO categories with the highest number of gene association in non-neurons. A bar graph was plotted with the top nodes based on the highest number of genes associated with a specific GO term. The y-axis shows the top 24 GO terms for the molecular function, while the x-axis shows the corresponding percentage of genes. The nodes with significant *p*-values beyond the 25th category were too low to be plotted. The specific values for the 24 nodes can be found in the additional file 21. (JPG 48.4 kb)

Additional file 21: Table S2. List of GO terms and their corresponding values. Table showing the top GO terms in the three categories; biological processes, molecular function and cellular components in both the cell populations (neuronal and non-neuronal) with their corresponding *p*-value as well as the percentage of genes associated from the data set. The values have been plotted and the bar graphs can be seen in the additional files 4, 5, 7, 8, 10, 11, 13, 14, 16, 17, 19, 20. (XLSX 27.9 kb)

Additional file 22: Table S3. List of molecular functions and their associated values for the present dataset. Table of all the values of the molecular functional enrichment analysis performed on the dataset. The analysis was accomplished with 1133 genes. The table gives the number of “molecules” (genes) associated with the particular molecular function as well as the percentage of genes that was used to create the pie charts (figure 5) in both the intestinal cell populations. (XLSX 37.7 kb)

Additional file 23: Table S4. List of the canonical pathways in the two intestinal cell populations. Pathway analysis on the genes enriched in both the populations to identify the most significant pathways. The analysis was accomplished using 1133 genes. The table provides the top canonical pathways with their respective –log(*p*-value) and the genes from the dataset that are known to be associated with that pathway. (XLSX 38.4 kb)

## Abbreviations

bp: pase pair
d.p.f: days post, fertilization
DEG: Differentially expressed genes
ENS: Enteric nervous system
FACS: Fluorescence, activated cell sorting
FDR: False discovery rate
FPKM: Fragments Per Kilobase of transcript per Million mapped reads
GFP: Green fluorescent protein
GFP + ve: Green fluorescent protein positive
GFP-ve: Green fluorescent protein negative
GI: Gastrointestinal
GO: Gene ontology
GTP: Gene transfer format
GWAS: Genome wide association studies
h.p.f: hours post fertilization
HSCR: Hirschprungs
IPA: Ingenuity pathway analysis
NCC: Neural crest cells
qPCR: Quantitative polymerase chain reaction
