# Supplementary figures and images for: Defining the transcriptomic landscape of the developing enteric nervous system and its cellular environment
Source: BMC Genomics. 2017 Apr 12;18:290. doi: 10.1186/s12864-017-3653-2 (PMC5389105; doi:10.1186/s12864-017-3653-2)

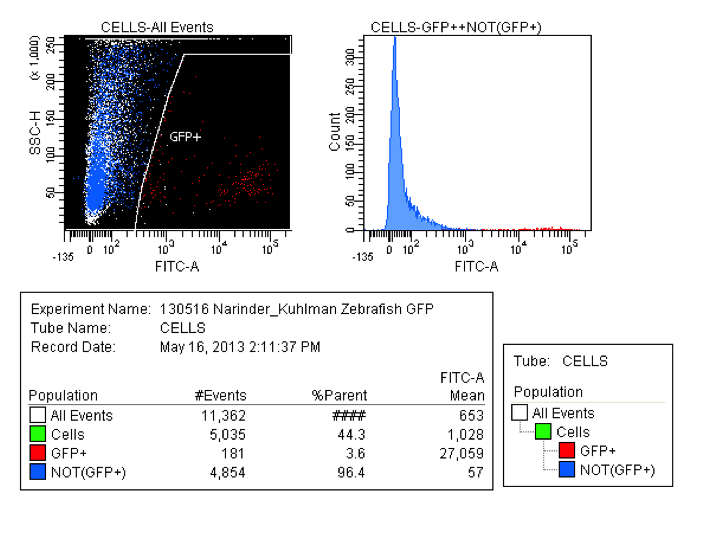

Supplement: Supplementary file 1 — Fluorescent Activated Cell Sorting (FACS) of the dissociated zebrafish. Fluorescent Activated Cell (FAC) analysis of sorted GFP-positive and GFP-negative cells. Viable cells separated into GFP-positive cells (red dots) and GFP-negative cells (blue dots). The table below gives an approximate survival and sorting rate of the sample. This particular analysis had 44.3% of live cells, among which 3.6% were positive and 96.4% were negative. (TIFF 1.48 mb) [file 12864_2017_3653_MOESM1_ESM.tiff]

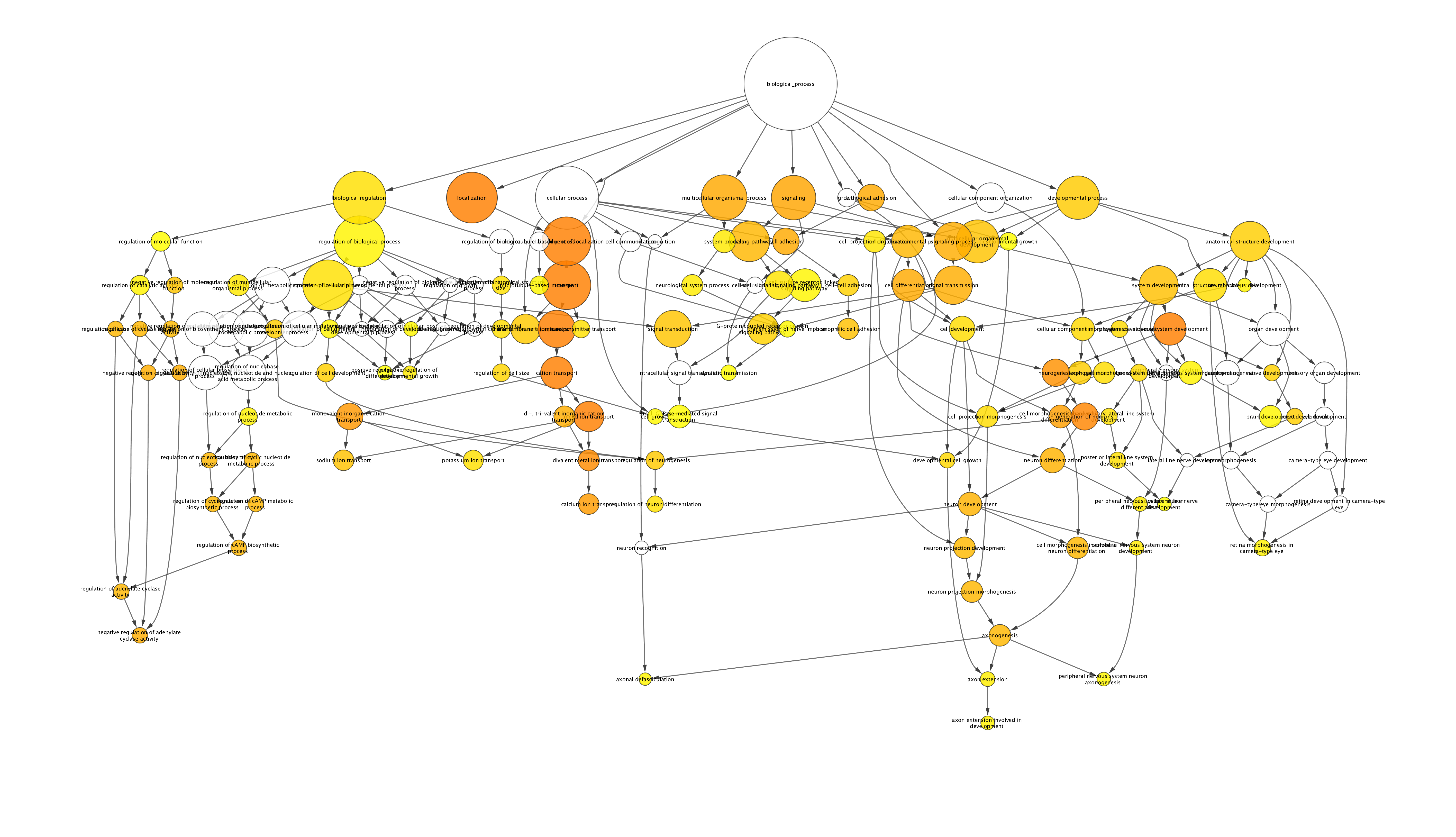

Supplement: Supplementary file 3 — The complete hierarchy showing the Biological processes category of GO enrichment in neurons. GO enrichment analysis of biological processes was performed with 530 genes which resulted in 143 enriched GO terms (nodes) and 227 edges. The size of the nodes refers to the number of genes associated with the GO term while the color represents the significance of association with the GO term. The low significant nodes to the higher significance ranges from white to dark orange nodes. (PNG 1.41 mb) [file 12864_2017_3653_MOESM3_ESM.png]

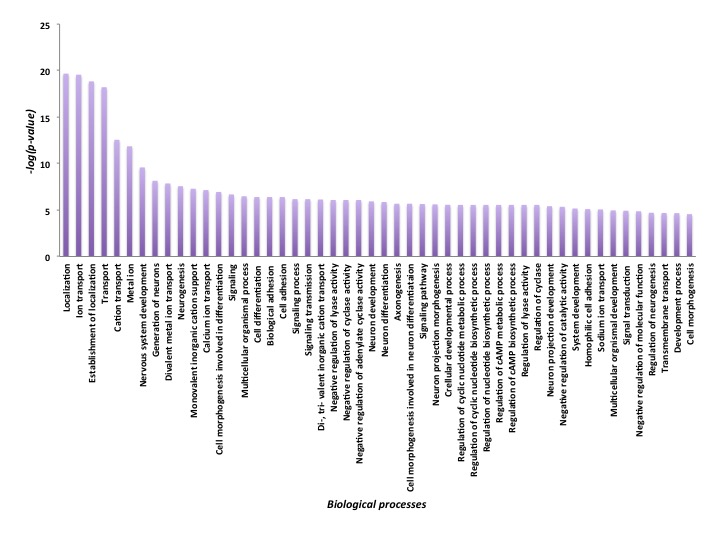

Supplement: Supplementary file 4 — Top 50 significant GO terms from the biological process category in the neurons. A bar graph illustrating the first significant 50 biological processes GO term nodes based on the p-value. The y-axis shows the top 50 GO terms for the biological processes, while the x-axis shows the corresponding –log(p-value). The specific values can be found in the additional file 21. (JPG 82 kb) [file 12864_2017_3653_MOESM4_ESM.jpg]

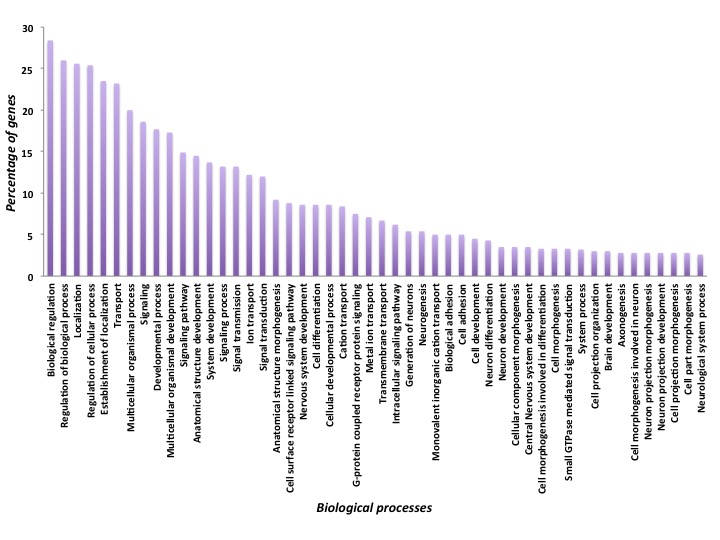

Supplement: Supplementary file 5 — Top 50 GO categories based on the highest percentages of genes associated with the GO terms. A bar graph illustrating the first 50 nodes based on the percentage of genes associated with a specific GO term. The y-axis shows the top 50 GO terms for the biological processes, while the x-axis shows the corresponding percentage of genes. The specific values can be found in the Additional file 21. (JPG 93.2 kb) [file 12864_2017_3653_MOESM5_ESM.jpg]

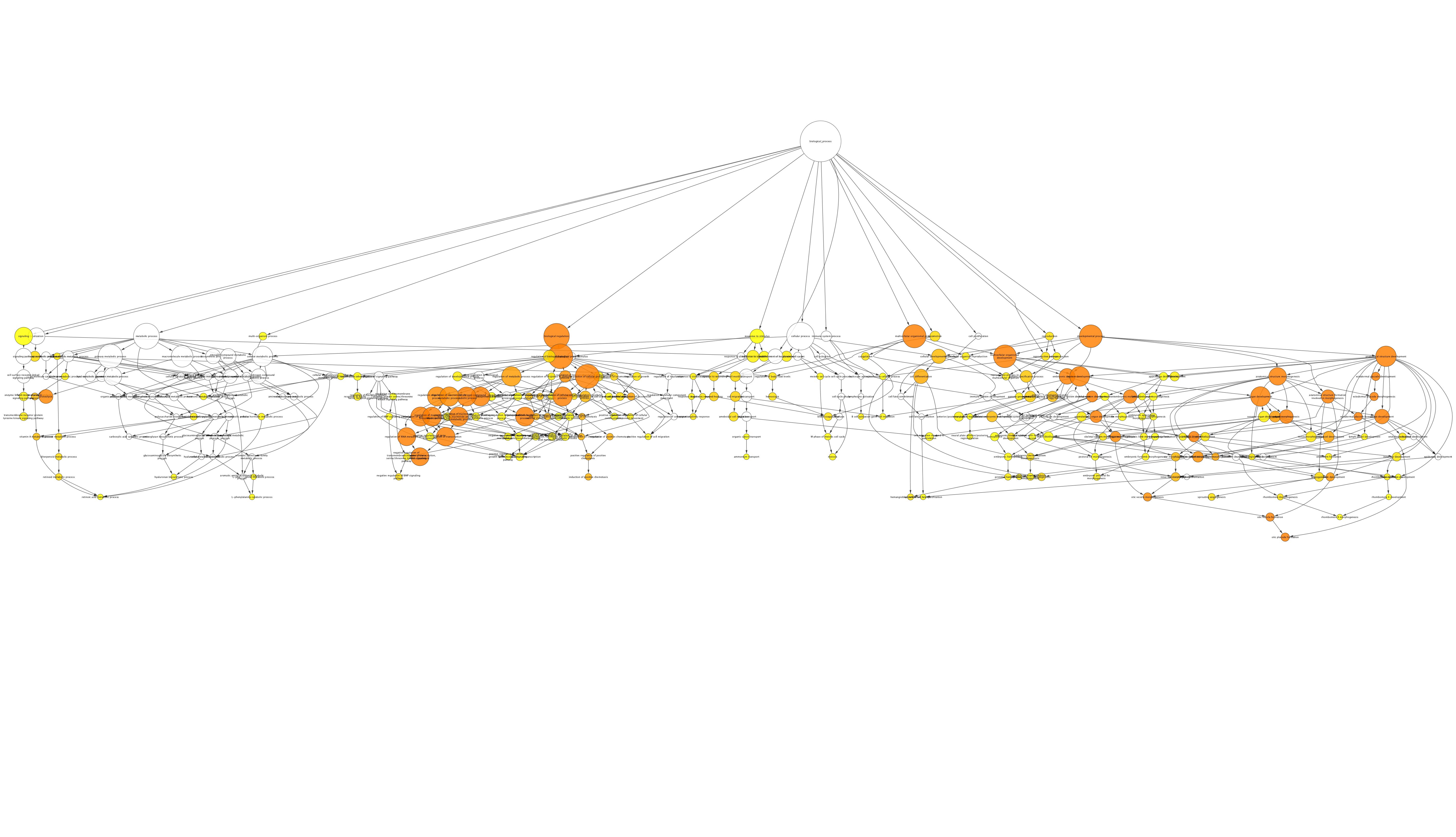

Supplement: Supplementary file 6 — The complete hierarchy showing the Biological processes category of GO enrichment in non-neurons. A GO enrichment analysis of biological processes in the non-neuronal genes. The analysis was performed with 482 genes which resulted in 280 enriched GO terms (nodes) and 467 edges. The size of the nodes refers to the number of genes associated with the GO term while the color represents the significance of association with the GO term. The low significant nodes to the higher significance ranges from white to dark orange nodes. (PNG 1.14 mb) [file 12864_2017_3653_MOESM6_ESM.png]

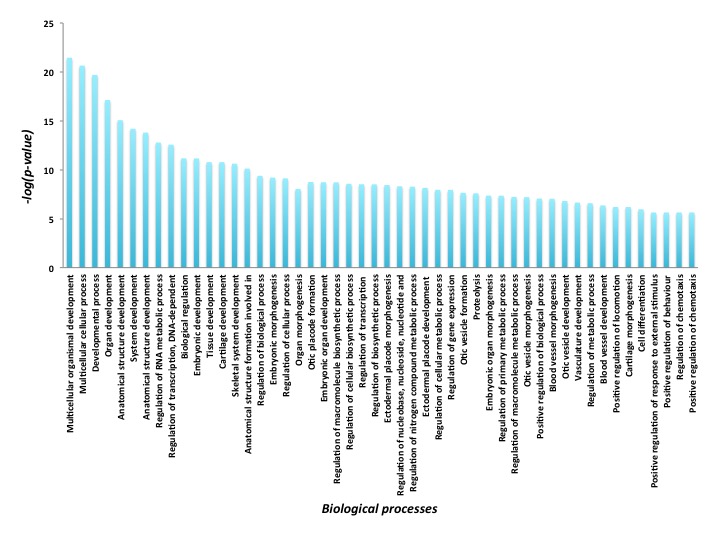

Supplement: Supplementary file 7 — Top 50 significant GO terms from the biological process category in the non-neurons. A bar graph plotted with the top 50 significant GO terms nodes based on the p-value. The y-axis shows the top 50 GO terms for the biological processes, while the x-axis shows the corresponding –log(p-value). The specific values can be found in the additional file 21. (JPG 97.7 kb) [file 12864_2017_3653_MOESM7_ESM.jpg]

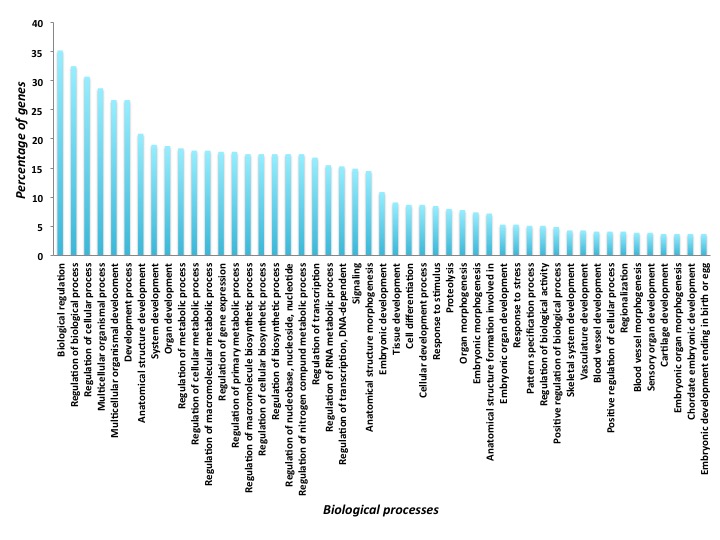

Supplement: Supplementary file 8 — Top 50 GO categories with the higher percentage of genes associated with a specific GO term in the non-neurons. A bar graph plotted with the first 50 nodes representing the highest percentage of genes associated with a specific GO term. The y-axis shows the top 50 GO terms for the biological processes, while the x-axis shows the corresponding percentage of genes. The specific values can be found in the additional file 21. (JPG 105 kb) [file 12864_2017_3653_MOESM8_ESM.jpg]

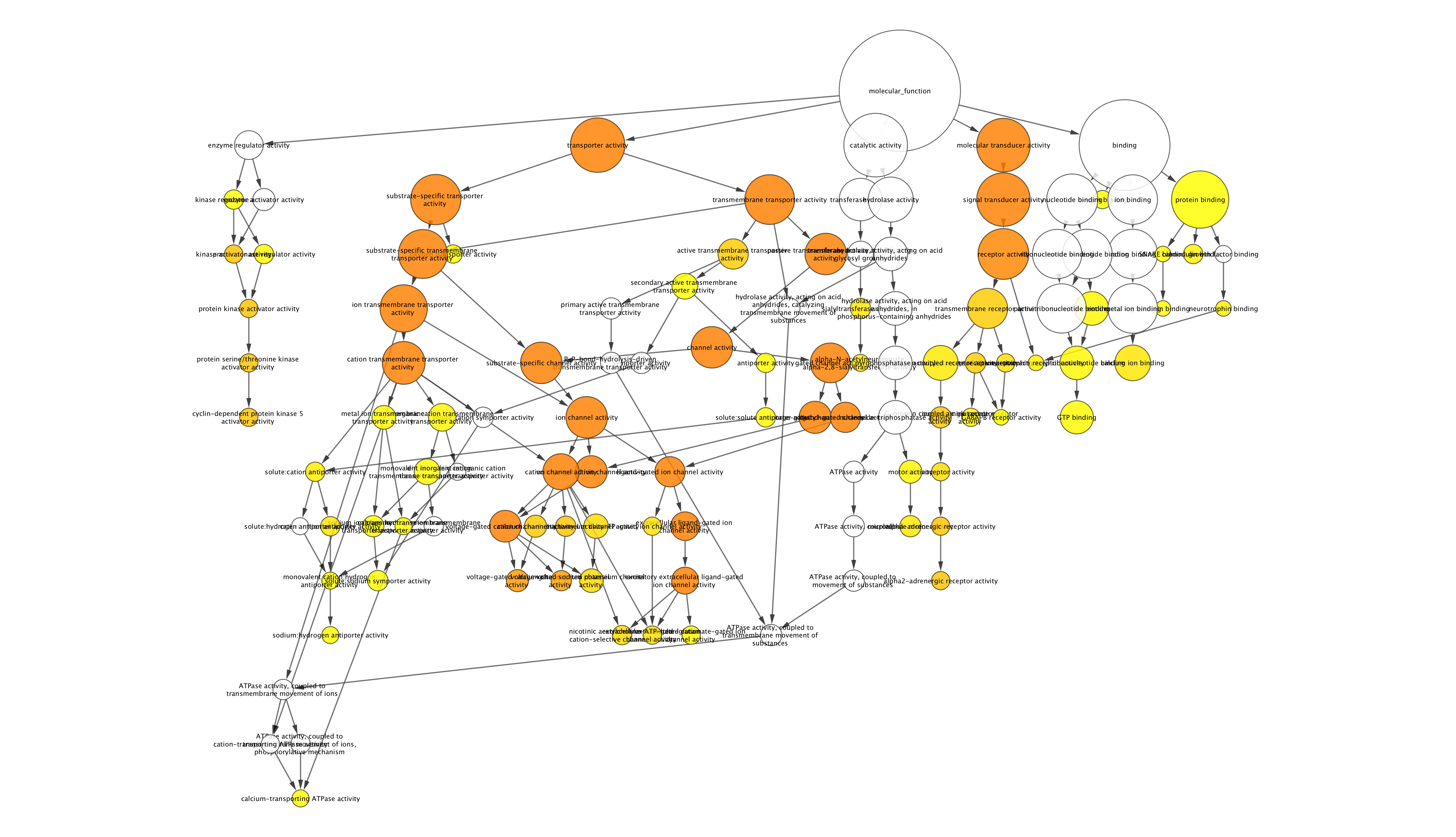

Supplement: Supplementary file 9 — The complete hierarchy showing the Molecular function category of GO enrichment in neurons. A GO enrichment analysis of specific molecular function the neuronal genes. The analysis was performed with 530 genes which resulted in 114 enriched GO terms (nodes) and 146 edges. The size of the nodes refers to the number of genes associated with the GO term while the color represents the significance of association with the GO term. The low significant nodes to the higher significance ranges from white to dark orange nodes. (PNG 1.26 mb) [file 12864_2017_3653_MOESM9_ESM.png]

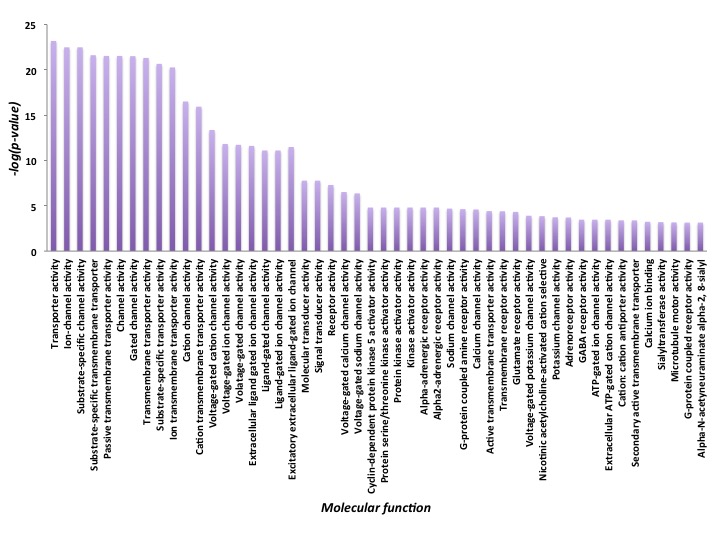

Supplement: Supplementary file 10 — Top 50 significant GO terms from the Molecular function category in neurons. A bar graph was plotted with the first 50 significant GO term nodes based on the p-value. The y-axis shows the top 50 GO terms for the molecular function, while the x-axis shows the corresponding –log(p-value). The specific values can be found in the additional file 21. (JPG 111 kb) [file 12864_2017_3653_MOESM10_ESM.jpg]

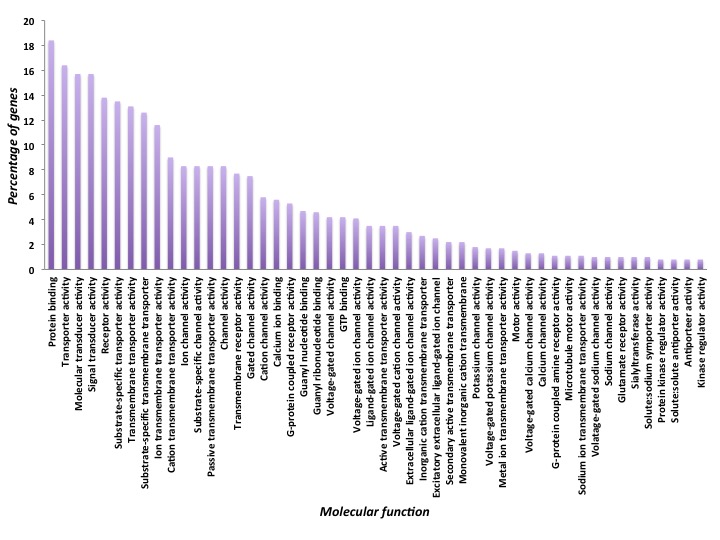

Supplement: Supplementary file 11 — Top 50 GO categories with the highest percentage of neuron associated genes. A bar graph plotted with the first 50 nodes based on the percentage of genes associated with a particular GO term. The y-axis shows the top 50 GO terms for the molecular function, while the x-axis shows the corresponding percentage of genes. The specific values can be found in the additional file 21. (JPG 106 kb) [file 12864_2017_3653_MOESM11_ESM.jpg]

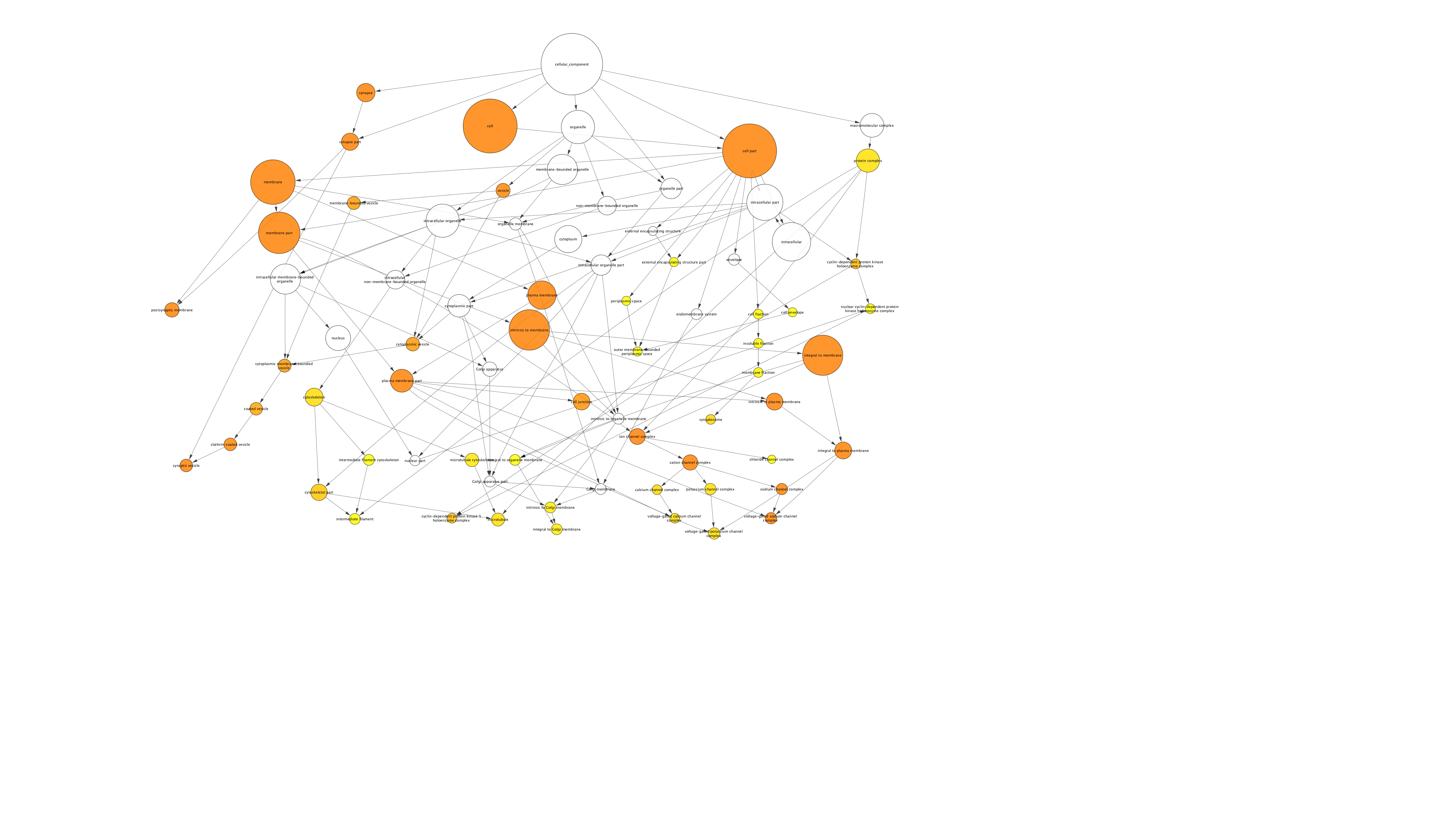

Supplement: Supplementary file 12 — The complete hierarchy showing the Cellular component category of GO enrichment in neurons. A graph of the cellular component GO term analysis of the neuronal genes. The analysis was performed with 530 genes which resulted in 75 enriched GO terms (nodes) and 126 edges. The size of the nodes refers to the number of genes associated with the GO term while the color represents the significance of association with the GO term. The low significant nodes to the higher significance ranges from white to dark orange nodes. (PNG 824 kb) [file 12864_2017_3653_MOESM12_ESM.png]

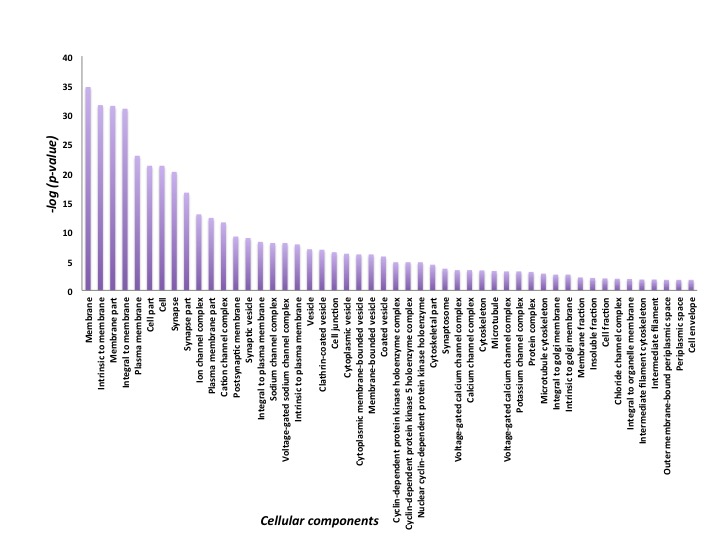

Supplement: Supplementary file 13 — Top 50 significant GO terms from the Cellular component category in neurons. A bar graph with the top 50 most significant nodes based on the p-value. The y-axis shows the top 50 GO terms for the cellular component, while the x-axis shows the corresponding –log(p-value). The specific values can be found in the additional file 21. (JPG 73 kb) [file 12864_2017_3653_MOESM13_ESM.jpg]

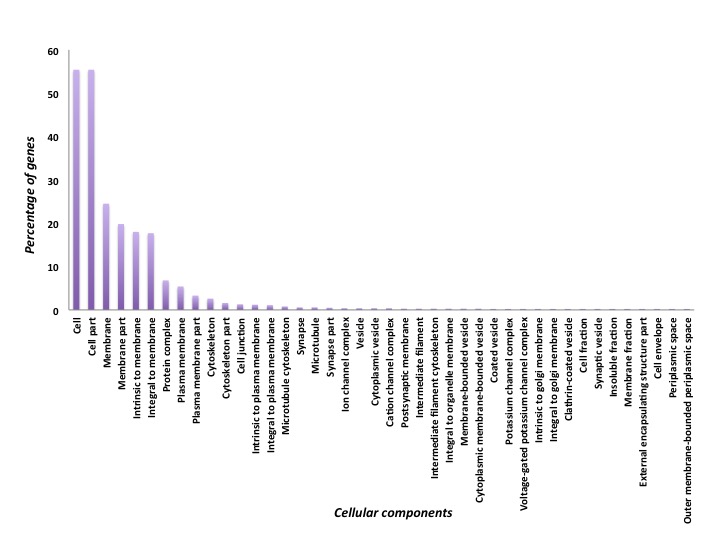

Supplement: Supplementary file 14 — Top GO categories with the largest number of gene association in neurons. A bar graph was plotted with the top nodes based on the percentage of genes associated with a particular GO term. The y-axis shows the top 41 GO terms for the molecular function, while the x-axis shows the corresponding percentage of genes. The small percentage of gene associated with GO terms beyond the 41st category were too low to be plotted. The specific values for the 41 nodes can be found in the additional file 21. (JPG 65 kb) [file 12864_2017_3653_MOESM14_ESM.jpg]

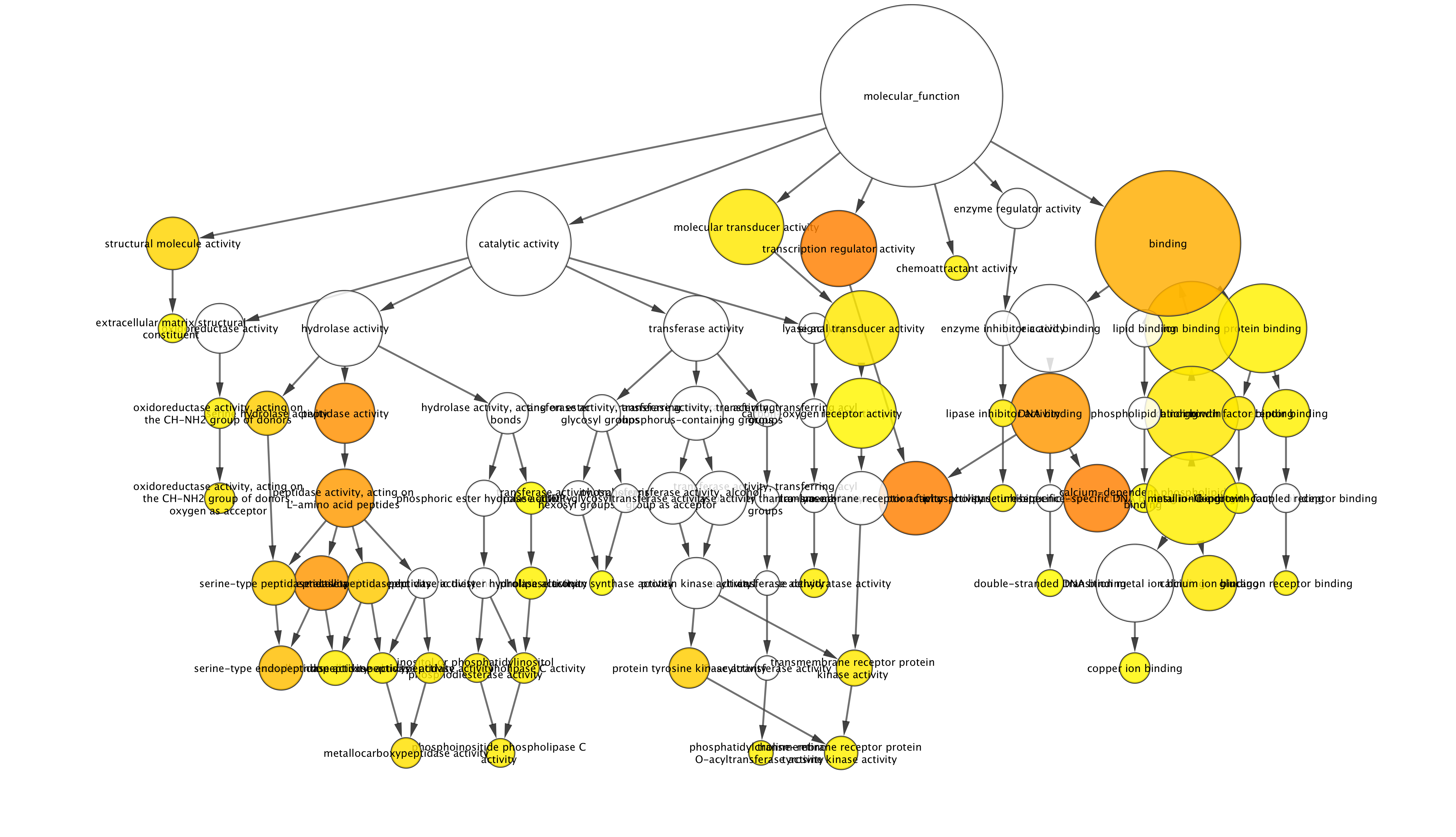

Supplement: Supplementary file 15 — The complete hierarchy showing the Molecular function category of GO enrichment in non-neurons. A GO enrichment analysis of molecular function in the non-neuronal genes. The analysis was performed with 482 genes which resulted in 81 enriched GO terms (nodes) and 92 edges. The size of the nodes refers to the number of genes associated with the GO term while the color represents the significance of association with the GO term. The low significant nodes to the higher significance ranges from white to dark orange nodes. (PNG 1.22 mb) [file 12864_2017_3653_MOESM15_ESM.png]

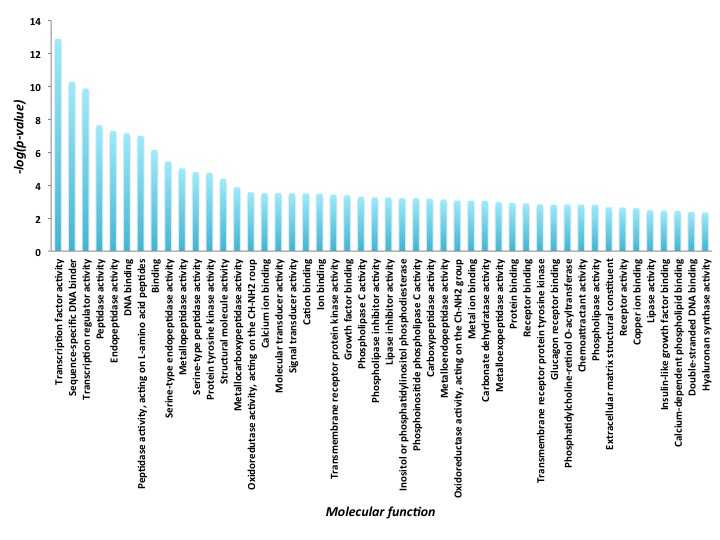

Supplement: Supplementary file 16 — Top 50 significant GO terms from the Molecular function category in the non-neurons. A bar graph was plotted with the top 50 significant GO term nodes based on the p-value. The y-axis shows the top 50 GO terms for the molecular function, while the x-axis shows the corresponding –log(p-value). The specific values can be found in the additional file 21. (JPG 97.3 kb) [file 12864_2017_3653_MOESM16_ESM.jpg]

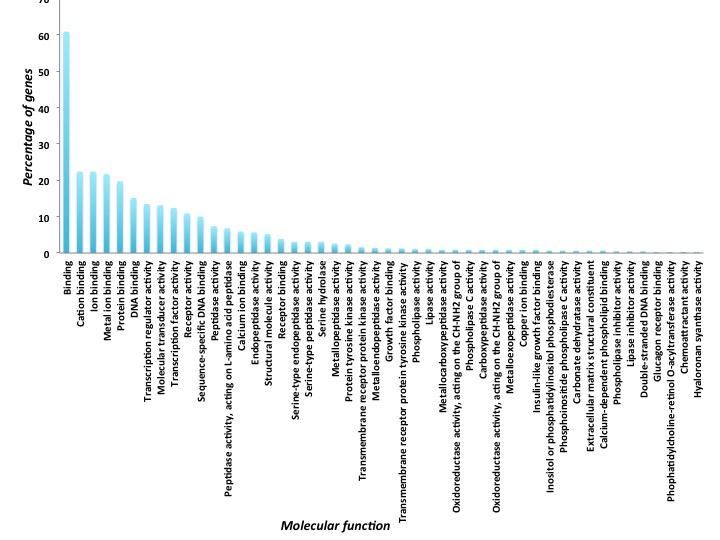

Supplement: Supplementary file 17 — Top 50 GO categories with the largest number of gene associated with GO terms in the non-neurons. A bar graph plotted with the top 50 nodes representing the highest percentage of genes associated with a specific GO term based on the percentage of genes associated. The y-axis shows the top 50 GO terms for the molecular function, while the x-axis shows the corresponding percentage of genes. The specific values can be found in the additional file 21. (JPG 88.5 kb) [file 12864_2017_3653_MOESM17_ESM.jpg]

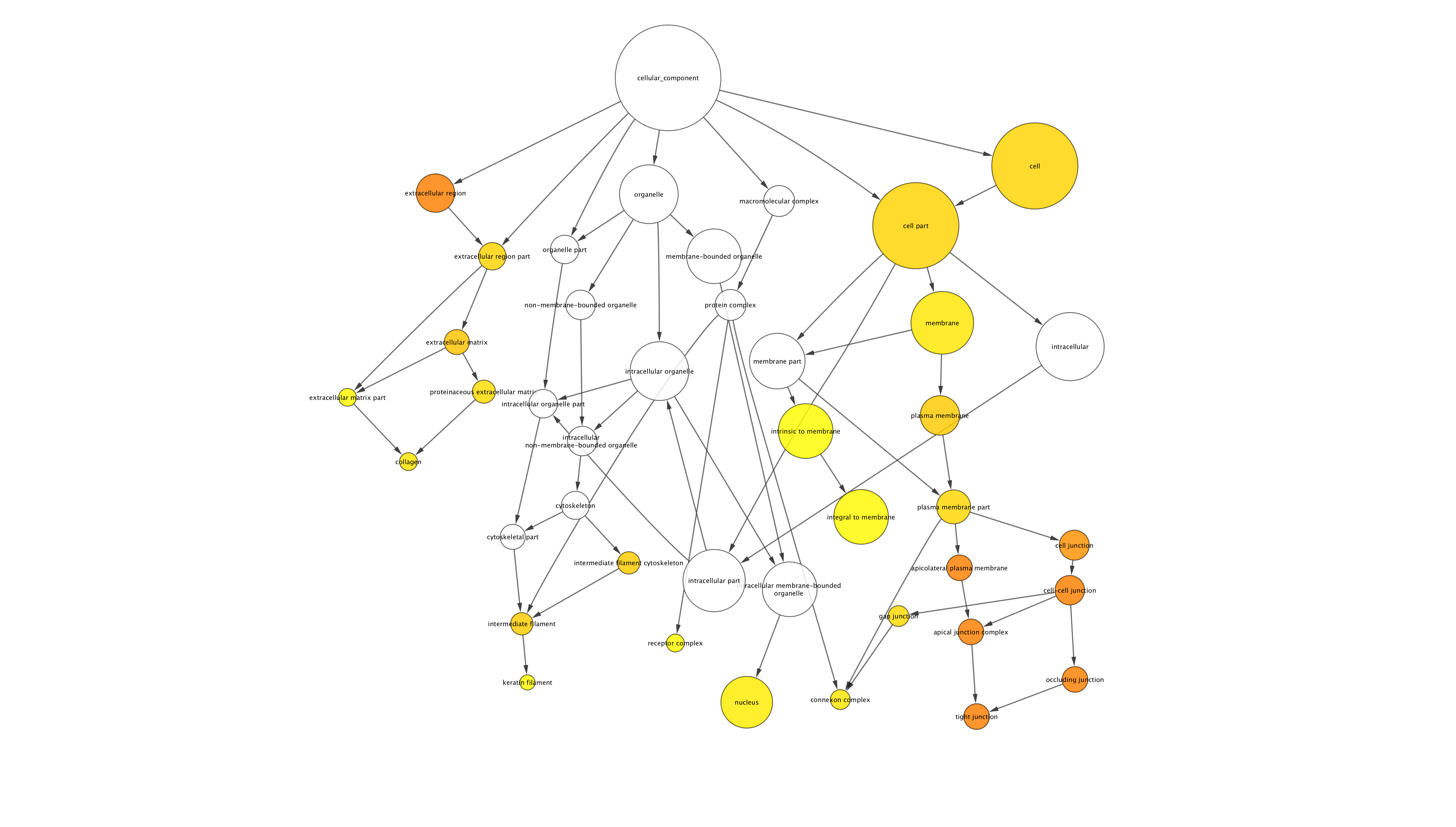

Supplement: Supplementary file 18 — The complete hierarchy showing the Cellular component category of GO enrichment in non-neurons. A GO enrichment analysis of cellular function in the non-neuronal genes. The analysis was performed with 482 genes which resulted in 42 enriched GO terms (nodes) and 61 edges. The size of the nodes refers to the number of genes associated with the GO term while the color represents the significance of association with the GO term. The low significant nodes to the higher significance ranges from white to dark orange nodes. (PNG 714 kb) [file 12864_2017_3653_MOESM18_ESM.png]

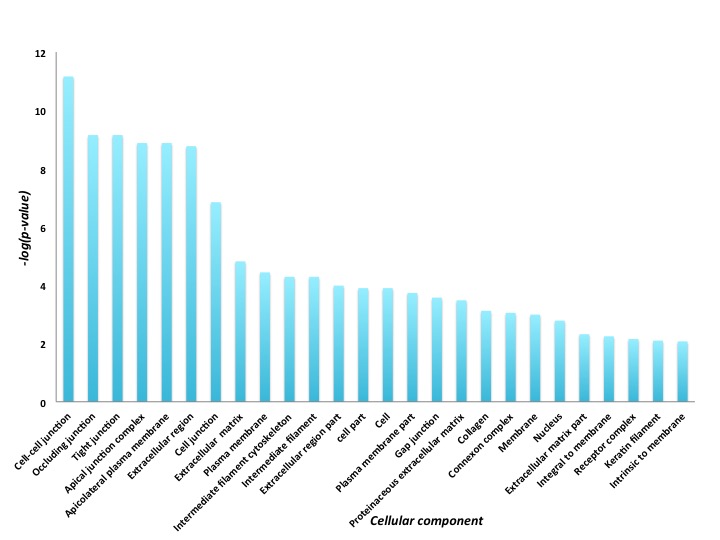

Supplement: Supplementary file 19 — Top significant GO terms from the Cellular component category in non-neurons. To identify and visualize some of the top significant GO terms, a bar graph was plotted with the top nodes based on the p-value. The y-axis shows the top 25 GO terms for the cellular component, while the x-axis shows the corresponding –log(p-value). The nodes with significant p-values beyond the 25th category were too low to be plotted. The specific values for the 25 nodes can be found in the additional file 21. (JPG 63.7 kb) [file 12864_2017_3653_MOESM19_ESM.jpg]

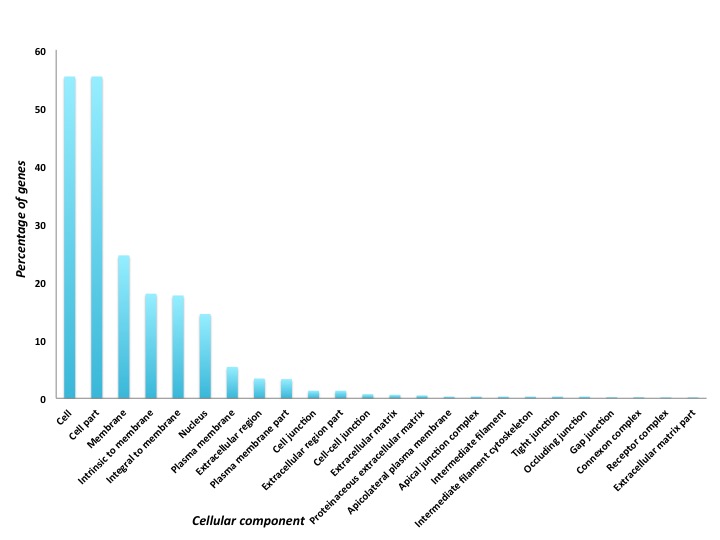

Supplement: Supplementary file 20 — Top GO categories with the highest number of gene association in non-neurons. A bar graph was plotted with the top nodes based on the highest number of genes associated with a specific GO term. The y-axis shows the top 24 GO terms for the molecular function, while the x-axis shows the corresponding percentage of genes. The nodes with significant p-values beyond the 25th category were too low to be plotted. The specific values for the 24 nodes can be found in the additional file 21. (JPG 48.4 kb) [file 12864_2017_3653_MOESM20_ESM.jpg]
